# Redox-reversible siderophore-based catalyst anchoring within cross-linked artificial metalloenzyme aggregates enables enantioselectivity switching†

**DOI:** 10.1039/d4cc01158a

**Published:** 2024-04-29

**Authors:** Alex H. Miller, Seán A. Thompson, Elena V. Blagova, Keith S. Wilson, Gideon Grogan, Anne-K. Duhme-Klair

**Affiliations:** a Department of Chemistry, University of York Heslington York YO10 5DD UK anne.duhme-klair@york.ac.uk; b Structural Biology Laboratory, Department of Chemistry, University of York Heslington York YO10 5DD UK

## Abstract

The immobilisation of artificial metalloenzymes (ArMs) holds promise for the implementation of new biocatalytic reactions. We present the synthesis of cross-linked artificial metalloenzyme aggregates (CLArMAs) with excellent recyclability, as an alternative to carrier-based immobilisation strategies. Furthermore, iron-siderophore supramolecular anchoring facilitates redox-triggered cofactor release, enabling CLArMAs to be recharged with alternative cofactors for diverse selectivity.

## Abstract 2

The cross-linking of redox-reversible artificial metalloenzyme aggregates produces recyclable protein microparticles that enable catalyst replacement and hence catalyst-controlled enantioselectivity switching.

## Graphical abstract

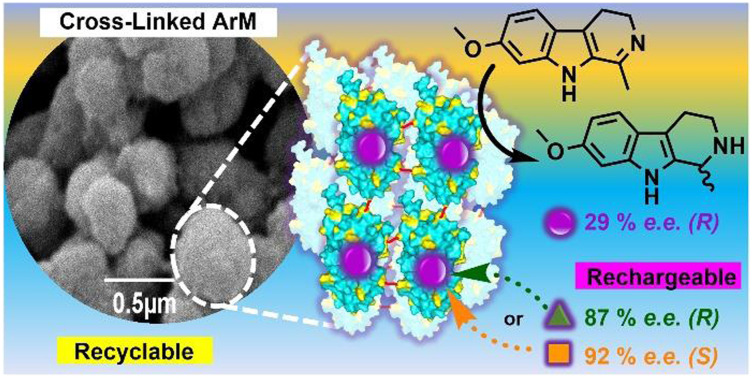

Artificial metalloenzymes aim at combining the broad reaction scope of metal complex catalysis with the selectivity and biocompatibility of proteins by inserting synthetic metal-based cofactors into protein scaffolds.^1–5^ Notwithstanding remarkable progress in this field, thus far artificial metalloenzymes have not progressed into widespread use, mainly because they are challenging and expensive to produce and their components cannot be reclaimed and recycled.^3^ In particular, the challenges associated with the initial stages of ArM development pose a substantial hurdle^5^ and the applicability of many ArMs remained limited by their low activity and stability, even if high selectivity could be achieved through rational design or directed evolution.^3–5^ Hence, there are clear directions for technological advancements in the field.^4,6^ The development of scalable technologies, in particular, is essential to enable the widespread adoption of ArMs, by ensuring that the application of these enzymes becomes commercially viable.

A key advancement to achieving scalability relies on the immobilisation of ArMs, an approach that has facilitated the industrial-scale utilisation of natural enzymes.^7,8^ Methods for immobilising ArMs have so far mostly utilised approaches based on carrier materials, such as adsorption,^9^ entrapment^10^ or covalent attachment.^11,12^ The effectiveness of these enzyme immobilisation methods, however, is often restricted by mass transfer limitations that arise from the use of the solid support material. In addition, solid supports often reduce enzyme activity due to altered microenvironments, limited substrate accessibility, and conformational changes that reduce the catalytic efficiency of the enzyme.^13^ Similarly, affinity-based approaches have also been used to immobilise ArMs.^14,15^

We have recently reported an affinity-based approach to immobilise a histidine-tagged redox-reversible artificial metalloenzyme onto conventional immobilised metal affinity chromatography (IMAC) resins.^15^ Our system takes advantage of the reversible binding of a synthetic catalyst *via* an Fe^III^-siderophore anchoring strategy^2^ to the protein scaffold, thereby enabling catalyst release and the subsequent recovery of the individual components. This catch-and-release strategy successfully enabled the immobilisation of the ArMs and their direct assembly from crude cell lysates. The immobilisation, however, significantly reduced turnover frequencies (TOFs), by around 3.4-fold, when compared to free enzyme in solution.

Alternative strategies that rely on cross-linked enzyme crystals (CLECs) have been explored for the carrier-free immobilisation of ArMs.^16–18^ Nevertheless, significant challenges in obtaining crystals, coupled with the complexities associated with scaling-up (high costs, time-consuming protocols, *etc.*), present notable obstacles.^19^

To overcome the inherent challenges associated with conventional carrier-based or crystal-dependent immobilisation strategies, Schoevaart *et al.* developed cross-linked enzyme aggregates (CLEAs) in the early 2000s.^20^ CLEAs involve the agglomeration of enzymes, which can be induced by organic solvents, salts or non-ionic polymers, followed by treatment with cross-linking agents, such as glutaraldehyde.^20–22^ Alternatively, agglomeration-independent cross-linking of enzymes can be achieved *via* biorthogonal strategies,^23,24^ using nonstandard amino acids, or through isocyanide-based multi-component reactions.^19^ In view of the limitations that current approaches to ArM immobilisation share, and inspired by the advances made with CLEAs, we aimed to develop an alternative carrier-free immobilisation method for ArMs, from here onwards referred to as cross-linked artificial metalloenzyme aggregates (CLArMAs).

Here we report the immobilisation of our previously-reported artificial imine reductase Gst-1-ArM (Scheme 1(a)).^15^ Gst-1-ArM consists of an iridium-containing catalyst attached to an Fe^III^-azotochelin-based anchor (1), which binds strongly but reversibly to Gst, a periplasmic binding protein scaffold from *Geobacillus stearothermophilus*.^25^ Gst-1-ArM agglomerates in the presence of organic solvents or ammonium sulfate (Scheme 1(b)), and the high density of lysine residues on the surface of Gst (Scheme 1(a)) enabled subsequent cross-linking with glutaraldehyde (Scheme 1(c)). The CLArMA synthesis was carried out by adapting the methods established for CLEAs.^20,21^ Since either excessive or insufficient cross-linking can compromise the formation of CLArMAs, finding the appropriate agglomeration and cross-linking conditions is key to achieving good immobilisation yields and activity recoveries.^21^

**Scheme 1:**
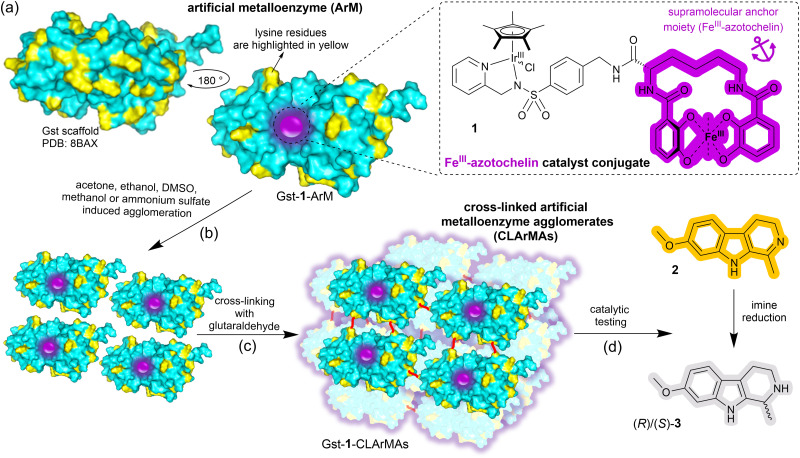
(a) Surface representation of the artificial metalloenzyme Gst-1-ArM with lysine residues highlighted in yellow and the anchored catalyst (1, shown in the inset) represented by a purple sphere. (b) Gst-1-ArM agglomeration triggered by organic solvents or ammonium sulfate. (c) Cross-linking of surface-exposed lysine residues with glutaraldehyde (red lines) to form cross-linked artificial metalloenzyme aggregates (CLArMAs). (d) Harmaline, 2, the substrate in the catalytic screening of the synthesised CLArMAs and formation of (*R*)/(*S*)-3.

Hence, a range of solvents was examined for the agglomeration and cross-linking of pre-assembled Gst-1-ArM (Table S1, entries 1–15, ESI†). In preliminary screening experiments, centrifugation speeds of up to 13 000 rpm proved inadequate for sufficient recovery of the formed Gst-1-CLArMA particles. Instead, centrifugal 50 kDa cut-off filters were used to isolate the formed particles, whilst remaining free ArMs (∼32 kDa) were not retained. The retrieved Gst-1-CLArMAs were then assessed for the reduction of the prochiral imine 2 to (*R*)/(*S*)-3 (Scheme 1(d)), and compared with free Gst-1-ArM in solution (Table S1, entry 0, ESI†). Whilst the enantioselectivity towards (*R*)-3 was maintained in all cases, ∼29% enantiomeric excess (e.e.), catalytic activities were agglomeration method dependent, with ammonium sulfate and methanol initially performing best. Subsequent tests identified ammonium sulfate as the agglomerant of choice, as the higher proportion of methanol that was necessary for sufficient agglomeration resulted in a reduction in both recovered activity and selectivity (Table S1, entries 16–19, ESI†).

A second round of optimisation using ammonium sulfate only, (Table S2, ESI†), endorsed the use of 70% ammonium sulfate saturation (2.87 M), 1-hour agglomeration, 2-hour cross-linking with 0.2% glutaraldehyde at 4 °C, and 400 rpm shaking (Table S2, entry 14, ESI†).

By using these optimised conditions, larger batches of Gst-1-CLArMAs were prepared and characterised. The immobilisation yields, solely based on the scaffold (84 ± 1%, Fig. S1, ESI†), are promising; however, only 77 ± 6% of the anticipated iridium level was detected in the recovered particles (ICP-OES, Table S3, ESI†). Partial detachment of the catalyst during agglomeration and cross-linking offers a plausible explanation for the iridium losses in the final samples. With a *K*_d_ value of 9.9 ± 0.9 nM,^15^ Gst has only a moderate binding affinity for 1. Therefore, the immobilisation yield, based on catalytic centres, is approximately 65%. Scanning electron microscopy images (Fig. 1(a)) show spherical particles with a diameter of approximately 0.5 μm. A rough surface and some porosity can be observed. The morphology of the Gst-1-CLArMA particles closely resembles that reported for type I CLEAs.^19,20^ The size distribution was confirmed by dynamic light scattering (Fig. S2, ESI†), with an average diameter of 0.71 μm.

**Fig. 1:**
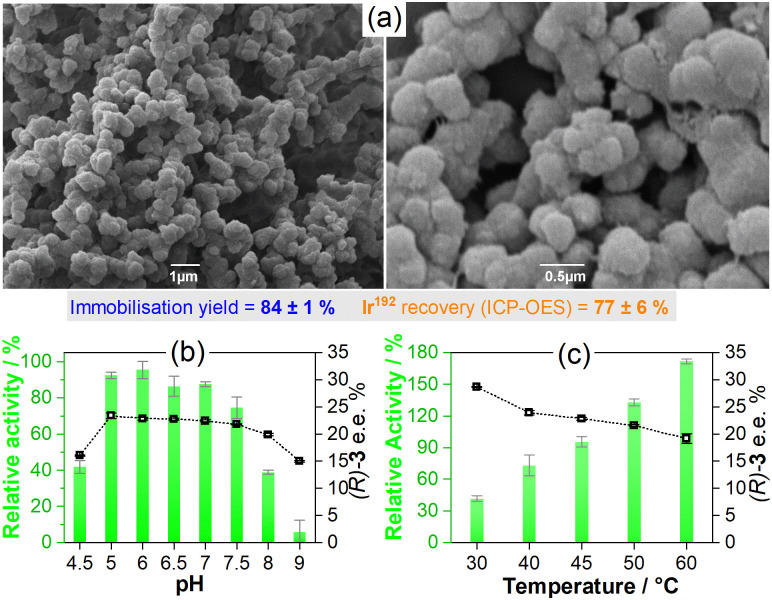
(a) SEM images of Gst-1-CLArMAs at 10 000× (left) and 30 000× (right) magnification. (b) Catalytic performance of Gst-1-CLArMAs for the reduction of 2 to (*R*)/(*S*)-3 achieved between pH 4.5 and 9 (45 °C, 400 rpm). (c) Catalytic performance at temperatures between 30 and 60 °C (pH 6, 400 rpm). Relative activities are represented by green columns and enantiomeric excesses in favour of the (*R*)-3 enantiomer are represented by open squares. Substrate concentration: 2 mM. Catalyst concentration: 20 μM (1 mol%, based on iridium level). Catalytic buffer: 0.6 M MES/3 M HCOONa. Error bars show the standard deviation based on triplicate measurements.

Gst-1-CLArMAs are stable between pH 5.0 and 7.5 and maintain their selectivity, as illustrated in Fig. 1(b). This represents a significant advance over prior studies that utilised an IMAC resin as carrier material for immobilisation,^15^ in which both free and immobilised ArM were only stable between pH 6 and 7. The formed Gst-1-CLArMAs microparticles exihibited a near neutral zeta potential of 0.3 ± 3.4 mV (Fig. S3, ESI†), which favours particle–particle interactions and their concomitant agglomeration. Vigorous shaking was therefore required for appropriate dispersion. The protonation/deprotonation of surface-exposed amino acid residues can lead to changes in the electrostatic interactions of the microparticles. The protonation state of several residues is pH-dependent, which could, in turn, be the reason for the more pronounced decrease in activity at pH 4.5, 8, and 9. However, the marked reduction in selectivity, particularly at pH 9, also suggests partial unfolding and/or conformational changes in proximity to the cofactor. The impact of temperature on both activity and selectivity (Fig. 1(b)) adhered to the previously reported trends, where elevated temperatures increase activity and decrease selectivity.^15^

To assess the recovery and reusability of the immobilised ArM, recyclability tests were carried out over eight consecutive catalytic runs. The Gst-1-CLArMA particles were found to retain their catalytic activity remarkably well, with over 90% or the initial activity remaining in the eighth cycle (Fig. 2(a)). Importantly, the e.e. was preserved across all repeat recycles, indicating that the site-specific binding of the cofactor was preserved. These results are in line with advanced natural enzyme immobilisation approaches, in which cross-linking strategies were implemented.^23,24^ Notably, the TOFs achieved with Gst-1-CLArMAs (0.43 min^−1^, calculated in relation to the iridium content) show only a 1.3-fold decrease in comparison to free Gst-1 ArM in solution (TOF = 0.6 min^−1^). This represents a significant improvement over the previously-reported IMAC resin-supported Gst-1, where immobilisation in Ni-sepharose microbeads led to a 3.4-fold decrease in TOF.^15^

**Fig. 2:**
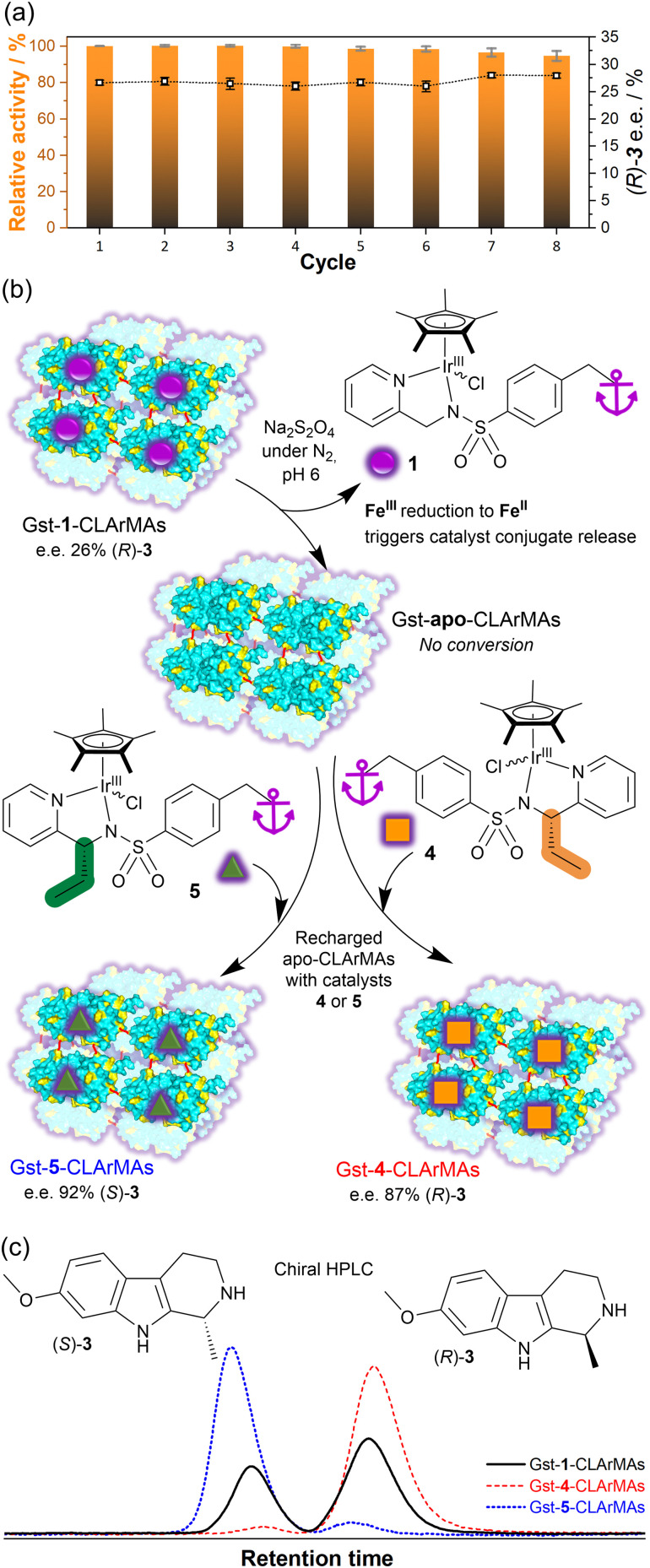
(a) Recycling tests with Gst-1-CLArMAs for the reduction of 2 to (*R*)/(*S*)-3 (pH 6, 45 °C, 800 rpm). Relative activities were calculated based on the activity measured in the first cycle. Enantiomeric excesses in favour of the (*R*)-3 enantiomer are represented by open squares. Substrate concentration: 2 mM. Catalyst concentration: 78 μM (4 mol%, based on iridium level). Catalytic buffer: 0.6 M MES/3 M HCOONa. (b) Schematic representation of the redox-triggered release of 1 (purple sphere) from Gst-1-CLArMAs, to form Gst-apo-CLArMAs, subsequently recharged with catalysts 4 (orange square) and 5 (green triangle). (c) Chiral HPLC traces of (*R*) and (*S*)-3 obtained for different CLArMAs under the same conditions as in the recycling tests outlined in (a).

Importantly, Gst-1 possesses a key feature that had yet to be explored in Gst-1-CLArMAs, namely the ability to release the inorganic catalyst from the protein scaffold upon chemical reduction of the Fe^III^-based anchor unit,^2,15^ thereby enabling either the replacement of catalyic centres that have lost activity with active centres or a switch to a different catalyst altogether.

To be able to assess the effect of protein cross-linking on reductive catalyst release and replacement, we synthesised two chiral ligands with opposite configurations (4, 5) to enable catalyst-controlled enantioselectivity switching, as outlined in Fig. 2(b) (ESI†: synthetic procedures, characterisation data, CD spectroscopic study of 1, 4, 5, Gst-1-, Gst-4- and Gst-5-ArMs).

In the first step, both fresh Gst-1-CLArMAs and Gst-1-CLArMAs that were recovered after the 8th recycling cycle, underwent a successful reduction step to remove 1, as evidenced by the absence of catalytic activity measured for the resulting cofactor-free “Gst-apo-CLArMAs”. Subsequently, the Gst-apo-CLArMAs were recharged with either catalyst 4 (recovered particles) and 5 (fresh particles), to enable a measurable switch in enantioselectivity. Due to its chiral center 4 produces (*R*)-3 with an e.e. of 77%, which increases to 95% after insertion into the Gst scaffold. With the recharged Gst-4-CLArMA particles, an e.e. of 87% in favour of (*R*)-3 was achieved, thereby confirming the successful incorporation of the artificial chiral cofactor. Catalyst 5, on the other hand, produces (*S*)-3 with an e.e. of 87%, which increases to 90% after insertion into the Gst scaffold, and increasing to 92% in the recharged Gst-5-CLArMA particles (Table S4, ESI†). Remarkably, the same cross-linked scaffold particles could be utilised to assemble three distinct artificial metalloenzymes, as evidenced by clear changes in enantioselectivity, illustrated in Fig. 2(c).

In summary, we have successfully demonstrated the synthetic viability of CLArMAs. This ArM immobilisation technique resulted in less than 30% loss in activity compared to the free ArM, marking a significant improvement over our previous carrier-based approach,^15^ in which a 70% activity reduction was observed. Moreover, Gst-1-CLArMAs exhibited excellent recyclability, retaining over 90% of their initial catalytic activity through to the 8th recycling cycle. Importantly, the redox-triggered disassembly feature remained intact, allowing catalyst switching. CLArMAs particles could be recharged with catalysts that gave rise to varied enantioselectivity in the reduction of harmaline. Artificial imine reductases with Cp*Ir-based artificial cofactors tend to show promiscuous catalytic activity prior to genetic optimisation.^26^ The Gst-1-ArM, for example, was previously used for the reduction of dehydrosalsolidine to (*R*)/(*S*)-salsolidine, whilst immobilised on a carrier resin.^15^ Since Gst-1-CLArMAs are likely to show similarly promiscuous behaviour, substrate scope investigations are currently underway. The development of CLArMAs that incorporate our siderophore-based catch-and-release approach to catalyst anchoring has opened up new avenues for advancing ArMs towards practical applications by offering a number of key benefits. Firstly, the new approach reduces the overall costs associated with ArM production and catalysis by extending their lifetime and facilitating the separation and recycling of components. Moreover, CLArMAs serve as a promising alternative to carrier-based and crystal-dependent immobilisation methods. By addressing issues such as mass transfer limitations and the time consuming, limited scalability of crystallisation protocols, they enhance efficiency and effectiveness.

## Conflicts of interest

There are no conflicts to declare.

## Supplementary Material

CC-060-D4CC01158A-s001
