# Supplementary material for: Redox-reversible siderophore-based catalyst anchoring within cross-linked artificial metalloenzyme aggregates enables enantioselectivity switching
Source: Chem Commun (Camb). 2024 Apr 29;60(42):5490–3. doi: 10.1039/d4cc01158a (PMC11107959; doi:10.1039/d4cc01158a)
Supplement: CC-060-D4CC01158A-s001 [file CC-060-D4CC01158A-s001.pdf]

Electronic Supplementary Material (ESI) for Chemical Communications.
This journal is © The Royal Society of Chemistry 2024

Redox-reversible siderophore-based catalyst anchoring within cross-linked artificial metalloenzyme aggregates enables enantioselectivity switching

Alex H. Miller,*[a] Seán A. Thompson,[a,b] Elena V. Blagova,[b] Keith S. Wilson, [b] Gideon Grogan [b] and Anne-K. Duhme-Klair *[a]

[a] Department of Chemistry, University of York, Heslington, York, YO10 5DD, United Kingdom

[b] Structural Biology Laboratory, Department of Chemistry, University of York, Heslington, York, YO10 5DD, United Kingdom

* Corresponding authors

Table of Contents

1. GENERAL INFORMATION 4
1.1. Instrumentation 4
1.2. Materials. 4
2. EXPERIMENTAL SECTION 5
2.1. ArM Assembly 5
2.2. Synthesis of CLArMAs 5
2.2.1. Preliminary screening procedure 1 5
2.2.2. Preliminary screening procedure 2 6
2.2.3. General optimised protocol for the synthesis of Gst-1-CLArMAs 9
2.3. Scanning Electron Microscopy 10
2.4. Dynamic Light Scattering and Zeta Potential 10
2.5. Gst-1-CLArMAs pH and temperature stability 11
2.6. Chiral HPLC method for compounds (*R*)-(+)-tetrahydroharmine and (*S*)-(–)-tetrahydroharmine 12
2.7. Recycling studies 12
2.8. Synthesis of (*S*)-4-cyano-*N*-(1-(pyridin-2-yl)propyl)benzenesulfonamide 13
2.9. Synthesis of (*S*)-4-(aminomethyl)-*N*-(1-(pyridin-2-yl)propyl)benzenesulfonamide 15
2.10. Synthesis of *N*,*N*'-((*S*)-6-oxo-6-((4-(*N*-((*S*)-1-(pyridin-2-yl)propyl)sulfamoyl)benzyl)amino)hexane-1,5-diyl)bis(2,3-bis(benzyloxy)benzamide) 18
2.11. Synthesis of *N*,*N*'-((*S*)-6-oxo-6-((4-(*N*-((*S*)-1-(pyridin-2-yl)propyl)sulfamoyl)benzyl)amino)hexane-1,5-diyl)bis(2,3-dihydroxybenzamide) 21
2.12. Synthesis of (*R*)-4-cyano-*N*-(1-(pyridin-2-yl)propyl)benzenesulfonamide 24
2.13. Synthesis of (*R*)-4-(aminomethyl)-*N*-(1-(pyridin-2-yl)propyl)benzenesulfonamide 27
2.14. Synthesis of *N*,*N*'-((*S*)-6-oxo-6-((4-(*N*-((*R*)-1-(pyridin-2-yl)propyl)sulfamoyl)benzyl)amino)hexane-1,5-diyl)bis(2,3-bis(benzyloxy)benzamide) 30
2.15. Synthesis of *N*,*N*'-((*S*)-6-oxo-6-((4-(*N*-((*R*)-1-(pyridin-2-yl)propyl)sulfamoyl)benzyl)amino)hexane-1,5-diyl)bis(2,3-dihydroxybenzamide) 33
2.16. CD-spectroscopic investigation of 1, 4, 5, Gst-1, Gst-4, Gst-5 36
2.17. Reductive release of the catalyst and reassembly of the CLArMAs 37
3. AUTHOR CONTRIBUTIONS 39
4. REFERENCES 39

1. GENERAL INFORMATION

The protein scaffold, *Gst*CeuE, shortly referred to as Gst, was expressed and purified as previously described.[1] The synthesis of compound **1** was performed in accordance to reported protocols,[2] adapted to form a 2.174 mM solution in DMF and MOPs (pH 7.5) mixture.[3] Compounds **4** and **5** were prepared using the same procedure,[3] using the precursors described herein: *N*,*N*'-((*S*)-6-oxo-6-((4-(*N*-((*S*)-1-(pyridin-2-yl)propyl)sulfamoyl)benzyl)amino)hexane-1,5-diyl)bis(2,3-dihydroxybenzamide) and *N*,*N*'-((S)-6-oxo-6-((4-(*N*-((*R*)-1-(pyridin-2-yl)propyl)sulfamoyl)benzyl)amino)hexane-1,5-diyl)bis(2,3-dihydroxybenzamide), respectively.

1.1. Instrumentation

NMR spectra were recorded on Jeol EX and ES 400 MHz instruments (^{1}H NMR 400 MHz, ^{13}C NMR 101 MHz). Electrospray ionisation mass spectrometry (ESI-MS) was performed on a Bruker compact® TOF mass spectrometer. Infrared (IR) spectra were recorded using Perkin Elmer UATR Two FT-IR spectrometer in ATR mode, covering wavenumber range 4000-400 cm^{-1}. UV-vis spectra were recorded on a Shimadzu UV-1800 in a quartz cuvette (Starna scientific). HPLC measurements were performed on an Agilent 1200 infinity II quaternary system equipped with a 1260 Quaternary Pump G7111B, G7116A multicolumn thermostat, G7165A multiwavelength detector and G7129A Vialsampler using the specified eluent gradients. Dynamic light scattering and zeta potential data were recorded using a Zetasizer Nano (Malvern Instruments Ltd., Worcestershire, UK), equipped with capillary cells (DTS 1060). Inductively coupled plasma-optical emission spectroscopy (ICP-OES) was carried out on an Agilent 5100 spectrometer and analysis was performed in ICP Expert version 7.6.2.12331. The circular dichroism spectroscopic measurements were performed on a Jasco J-1500 CD Spectrophotometer at 20 °C under constant nitrogen flush.

1.2. Materials.

Unless otherwise noted, reagents were used as received from commercial suppliers and used as supplied. All expression media and buffers were prepared using ddH_2O (purification system, Millipore). Solvents for chromatography were HPLC grade. $[Ir(Cp^*)(Cl)_2]_2$ was prepared as described in the literature.[4] 1-Methyl-7-methoxy-3,4-dihydro-β-carboline (harmaline, ≥ 95 %), glutaraldehyde (25 % solution), glycine (≥ 98.5 %), ammonium sulphate ($(NH_4)_2SO_4$, ≥ 99 %), sodium phosphate monobasic ($NaHPO_4$), sodium phosphate dibasic (Na_2HPO_4), sodium hydroxide (NaOH) and ammonium hydroxide (NH_4OH) were purchased from Sigma-Aldrich, tris(hydroxymethyl)aminomethane hydrochloride (Tris-HCl, ≥ 99 %), Bradford dye reagent (ready-to-use solution) and low retention microcentrifuge tubes were purchased from ThermoFisher Scientific, Alfa Aesar.

2. EXPERIMENTAL SECTION

2.1. ArM Assembly

3120 nmol of artificial transfer hydrogenase, herein denoted Gst-**1**, was assembled in accordance with a previously established protocol,[3] albeit with minor adjustments. Specifically, 0.1 M phosphate buffer at pH 7 was employed during the concentration steps in order to buffer-exchange (in the previous protocol, 0.05 M Tris-HCl, 0.15 M NaCl buffer at pH 7.5 was used instead). The latest step was carried out to reduce the concentration of **tris**(hydroxymethyl)aminomethane (Tris) and prevent the quenching of glutaraldehyde when the cross-linking step is carried out. Subsequently, the final concentration of Gst-**1** was adjusted to 1 mM using 0.1 M phosphate buffer at pH 7. The resulting product was refrigerated (< 8 °C) for storage before subsequent use.

2.2. Synthesis of CLArMAs

2.2.1. Preliminary screening procedure 1

Acetone, methanol, ethanol and DMSO as well as ammonium sulfate, traditionally used in the synthesis of cross-linked enzyme aggregates,[5] were screened for the agglomeration of Gst-**1** and subsequent cross-linking with glutaraldehyde. Procedure: 250 µL of 0.04 mM Gst-**1** in 0.1 M phosphate buffer, pH 7, was transferred to a 2 mL low retention microcentrifuge tubes and subjected to incubation at 4 °C for 5 minutes on a thermos-shaker for microtubes (Grant-bio). Subsequently, 125, 250, 500 µL of ice-cold organic solvent or saturated ammonium sulfate solution (4.1 M at 25 °C) were slowly pipetted into the pre-incubated solution. Following this, 20, 27.5 or 40 µL of a 2% glutaraldehyde aqueous solution, freshly prepared by dilution from a 25% commercial stock, was introduced into the suspension from the previous step, so that the concentration of glutaraldehyde is fixed at 0.1% in all cases. The resulting mixture was agitated for 2 hours at 4 °C, with shaking at 250 rpm. Finally, to quench any unreacted aldehydes and prevent additional cross-linking in subsequent stages, 500 µL of a 0.2 M glycine aqueous solution was added to the mixture, which was then shaken for 30 min at 4 °C and 400 rpm. The Gst-**1**-CLArMAs were recovered through filtration using 50 kDa cut-off spin filters, for 5 min at 13000 rpm, 10 °C. Filtrates were washed twice with phosphate buffer, and centrifuged again. Final recovered aliquots (~25 µL) were transferred to a 2 mL microcentrifuge tube and tested for the imine reduction of **2**, as summarised below.

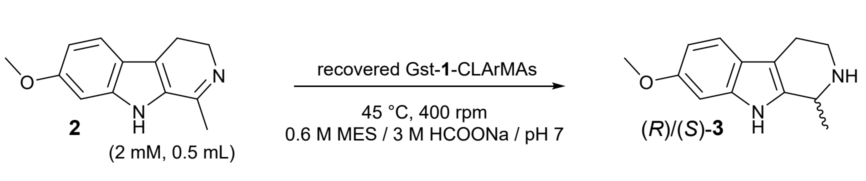

After 2 h, 50 µL of the reaction mixture were collected and quenched in 2 mL of 12.5 mM glutathione in a 2:1 MeOH:H_2O solution. The quenched reaction was transferred to a cuvette (1 cm), and UV-vis spectra were recorded to monitor intensity decay at 375 nm, reflecting imine consumption and amine

formation.[3] Subsequently, 1 mL of the quenched sample was filtered into an HPLC vial using a 0.22 µm Nylon syringe filter and stored for chiral chromatography analysis. General conditions tested, recovered activities and respective (*R*)-**3** e.e. % are summarised in Table S1.

Table S1. Screening conditions, recovered activity and e.e. for conditions screened using procedure 1.

**Entry**	**Solvent/salt** [a]	**Ratio ArM stock** [b] **: Solvent**	**Recovered activity** [c] **/ %**	**(*R*)-3 e.e.** [d] **/ %**
0	-	1:0	100	29
1	acetone	2:1	15.0 ± 0.1	29
2	methanol		43 ± 2	30
3	ethanol		43.0 ± 0.9	29.7 ± 0.3
4	DMSO		34 ± 2	28.8 ± 0.4
5	ammonium sulfate		51 ± 6	29.4 ± 0.1
6	acetone	1:1	12 ± 2	28.7 ± 0.2
7	methanol		42 ± 2	30.0 ± 0.1
8	ethanol		28 ± 3	29
9	DMSO		18 ± 6	29.6 ± 0.4
10	ammonium sulfate		44 ± 7	29
11	acetone	1:2	9 ± 4	30.0 ± 0.1
12	methanol		62 ± 1	30
13	ethanol		24.1 ± 1.5	29.6 ± 0.1
14	DMSO		4 ± 5	0
15	ammonium sulfate		35 ± 5	31
16	methanol	1:4 [e]	14 ± 4	31 ± 2
17	ammonium sulfate		60 ± 10	33 ± 2
18	methanol	1:8 [e]	0	-
19	ammonium sulfate		35 ± 3	31.9 ± 0.1

[a] agglomeration solvent/salt.
[b] Gst-**1**-ArM stock in phosphate buffer, pH 7. Agglomeration and cross-linking procedure:
[c] Recovery activity was calculated using a control with free Gst-**1**-ArM, entry 0, as reference.
[d] deviation below 0.1 was omitted and the mean value rounded.
[e] 50 µL of 0.2 mM Gst-**1** and 200 or 400 µL of ice-cold solvent/salt used instead. All other quantities and steps were carried out as described in procedure 1.

2.2.2. Preliminary screening procedure 2

Using saturated ammonium sulfate (4.1 M at 25 °C), pH 7, as agglomeration inducer, a second set of conditions was screened to investigate salt saturation, cross-linker percentage and cross-linking

duration. Procedure: 30 µL (or volume indicated in Table S2) of 1 mM Gst-**1** in 0.1 M phosphate buffer, pH 7, was transferred to a 2 mL low retention microcentrifuge tubes. Volumes of phosphate buffer, pH 7, were added to respective microtubes (Table S2), and the mixture subjected to incubation at 4 °C for 5 min on a thermos-shaker for microtubes (Grant-bio). Subsequently, volumes of saturated ammonium sulfate (Table S2), pH 7, were slowly pipetted into the pre-incubated solution, and subsequently left under incubation at 4 °C, 400 rpm for 1 h. Following this, 20 µL of a glutaraldehyde aqueous solution (% concentration indicated in Table S2), freshly prepared by dilution from a 25% commercial stock, was introduced into the suspension from the previous step. The resulting mixture was agitated for 2 hours at 4 °C, with shaking at 400 rpm. Finally, to quench any unreacted aldehydes and prevent additional cross-linking in subsequent stages, 80 µL of a 1 M glycine aqueous solution was added to the mixture, which was then shaken for 1 h at 21 °C and 400 rpm. The Gst-**1**-CLArMAs were recovered through centrifugation, for 5 min at 4000 rpm, 10 °C. Precipitates were washed twice with phosphate buffer, and centrifuged again. Final recovered aliquots (~25 µL) were transferred to a 2 mL microcentrifuge tube and tested for the imine reduction of **2**, as summarised below.

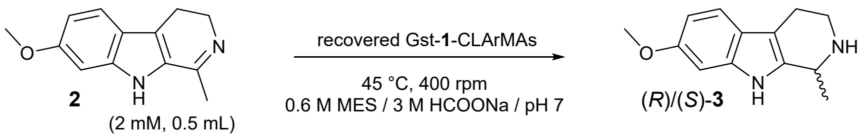

After 1 hour, 25 µL of the reaction mixture was collected and quenched in 2 mL of 12.5 mM glutathione in a 2:1 MeOH:H_2O solution. The quenched reaction was transferred to a cuvette (1 cm), and UV-vis spectra were recorded to monitor intensity decay at 375 nm, reflecting imine consumption and amine formation.[3] Subsequently, 1 mL of the quenched sample was filtered into an HPLC vial using a 0.22 µm Nylon syringe filter and stored for chiral chromatography analysis. General conditions tested, recovered activities and respective (*R*)-**3** e.e. % are summarised in Table S2.

Table S2. Screening conditions, recovered activity and e.e. % for conditions screened using procedure 2.

Entry	ArM stock / µL	Phosphate Buffer / µL	Saturated Ammonium Sulfate / µL	Glutaraldehyde (stock) / µL (%)	Glutaraldehyde / %	Cross-linking time / h	Recovered activity / %	(*R*)-3 e.e. [a] / %
0	30	-	-	-	-	-	100	27.5
1	30	110	260	20 (4.2 %)	0.2	2h	N/R	-
2	30	70	300	20 (4.2%)	0.2	2h	45 ± 2	15.6 ± 0.5
3	30	30	340	20 (4.2%)	0.2	2h	50 ± 1	14.6 ± 0.2
4	30	110	260	20 (4.2%)	0.2	2h	N/R	-
5	30	70	300	20 (4.2%)	0.2	2h	73 ± 1	17.3 ± 0.2
6	30	30	340	20 (4.2%)	0.2	2h	68 ± 2	16.0
7	30	70	300	20 (4.2%)	0.2	2h	82 ± 1	17.0
8	30	70	300	20 (3.15%)	0.15	2h	50	19.2 ± 0.5
9	30	70	300	20 (2.1%)	0.1	2h	N/R	-
10	30	70	300	20 (1.05%)	0.05	2h	N/R	-
11	30	70	300	20 (4.2%)	0.2	1.5 h	77 ± 2	17.5 ± 0.1
12	30	70	300	20 (4.2%)	0.2	2.5h	79 ± 1	17.1 ± 0.4
13	60	40	300	20 (4.2%)	0.2	2 h	69 ± 2	18.0
14	100	0	300	20 (4.2%)	0.2	2 h	61 ± 1	18.8 ± 0.1
15	60	40	300	20 (8.4%)	0.4	2 h	80 ± 3	15.3 ± 0.3

N/R – no particles recovered after centrifugation for 5 min at 4000 rpm nor at 13000 rpm. [a] deviation below 0.1 was omitted and the mean value rounded.

2.2.3. General optimised protocol for the synthesis of Gst-1-CLArMAs

After analysis of the results obtained using preliminary procedures 1 and 2, a general protocol was established. A total volume of 450 µL containing 1 mM Gst-**1** was transferred to a 2 mL low retention microcentrifuge tube and subjected to incubation at 4 °C for 5 min on a thermos-shaker for microtubes (Grant-bio). Subsequently, 1350 µL of saturated ammonium sulfate at pH 7 (adjusted with a 5 M NH_4OH solution) was pipetted into the pre-incubated solution. The resulting mixture was agitated for 1 hour at 4 °C, with shaking at 400 rpm, to induce aggregation. Following this, 90 µL of a 4.2% glutaraldehyde aqueous solution, freshly prepared by dilution from a 25% commercial stock, was introduced into the suspension from the previous step. The combined solution underwent further shaking for 2 hours at 4 °C and 400 rpm. Finally, to quench any unreacted aldehydes and prevent additional cross-linking in subsequent stages, 300 µL of a 1 M glycine aqueous solution was added to the mixture, which was then shaken for 1 hour at 21 °C and 400 rpm.

The Gst-**1**-CLArMAs were recovered through centrifugation at 4000 rpm for 5 min. The supernatant was carefully withdrawn using a pipette and reserved for subsequent determination of cross-linking yield using Bradford assay,[6] Fig. S1. The resulting precipitate underwent a thorough washing process with 1 mL of 0.05 M Tris-HCl, 0.15 M NaCl at pH 7 buffer, employing strong vortexing, and concluding with a centrifugation step at 4000 rpm for 5 minutes. This washing procedure was repeated once more with the same buffer and once again with 0.05 M MES/0.25 M HCOONa at pH 7 buffer, used for long term storage. The final recovered particles were resuspended in 0.05 M MES/0.25 M HCOONa at pH 7 buffer, with the volume adjusted to 1.5 mL.

Three distinct batches (1, 2 and 3) were synthesised and employed for statistical analysis in subsequent investigations. 30 µL of sample from each batch was submitted to ICP-OES analysis to estimate Ir^{192} recovery after cross-linking (Table S3).

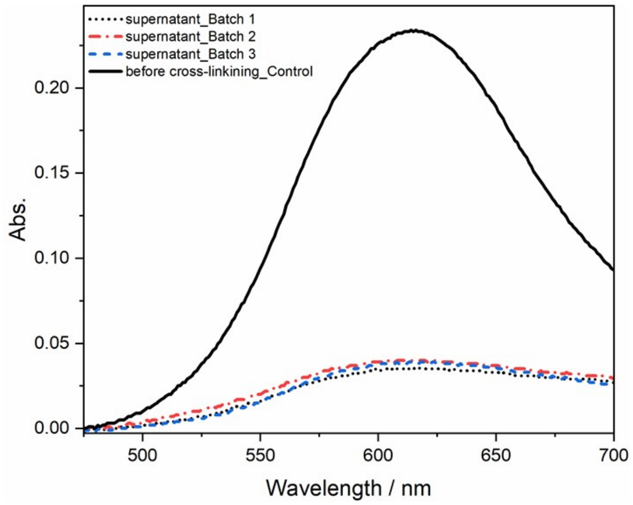

Fig. S1 UV-vis spectra from Bradford assays used to monitor Gst-**1** in the supernatant after Gst-**1**-CLArMAs recovery through centrifugation (batches 1, 2 and 3). Absorbance at 595 nm was used to estimate the immobilisation yields, compared to a control (sample before cross-linking). Procedure: 50-fold dilutions of all samples were prepared in 0.1 M phosphate buffer, pH 7. A 20 µL sample was mixed with 1200 µL of Bradford's reagent. After 10 min, UV-vis spectra were collected. Triplicate measurements were carried out for each sample. The background obtained using 0.1 phosphate buffer, and subtracted from all runs.

Table S3. ICP-OES

**Sample**	**Ir^{192} / ppm**	**Ir^{192} / mol [a]**	**Recovered Ir^{192} / % [b]**
Batch 1	0.212	6.04167×10^{-9}	79.91623
Batch 2	0.214	6.14583×10^{-9}	81.29409
Batch 3	0.197	5.26042×10^{-9}	69.58223
		**Mean ± S.D.**	**77 ± 6**

[a] The analysis blank (0.096 ppm) was deducted before calculation of total amount if Ir^{192}.
[b] Calculated using the concentration of Ir^{192} expected in case the total amount of protein determined through Bradford assay would have conserved the cofactor bound (7.56×10^{-9} mol) after cross-linking.

2.3. Scanning Electron Microscopy

Gst-**1**-CLArMAs in suspension underwent a washing and dehydration process as follows: distilled water three times; 100% ethanol three times; hexamethyldisilazane twice. Each wash/dehydration step lasted for ten minutes on a rotator, and the sample was pelleted between washes at 4000 *x g* for 5 minutes. The majority of hexamethyldisilazane was removed by pipetting, and the sample was left to dry overnight in a desiccator before mounting for SEM (Aluminium SEM stub and adhesive carbon tab). The sample was sputter-coated with gold/palladium before viewing in a Jeol 6490 SEM operating at 5kV.

2.4. Dynamic Light Scattering and Zeta Potential

Dynamic light scattering data were recorded using a Zetasizer Nano (Malvern Instruments Ltd., Worcestershire, UK). Data were recorded from 15–20 runs per single measurement, carried out at

25°C using folded capillary cells (DTS 1060). Solutions were freshly prepared by dissolving an appropriate amount of Gst-1-CLArMAs stock in filtered buffer at pH 6 (0.05 M MES/0.25 M HCOONa).

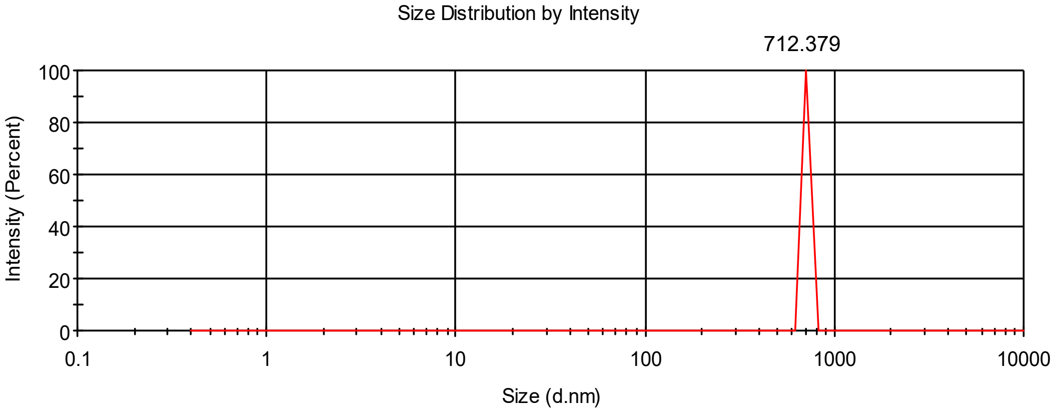

Fig. S2 Gst-**1**-CLArMAs size distribution by DLS measurements.

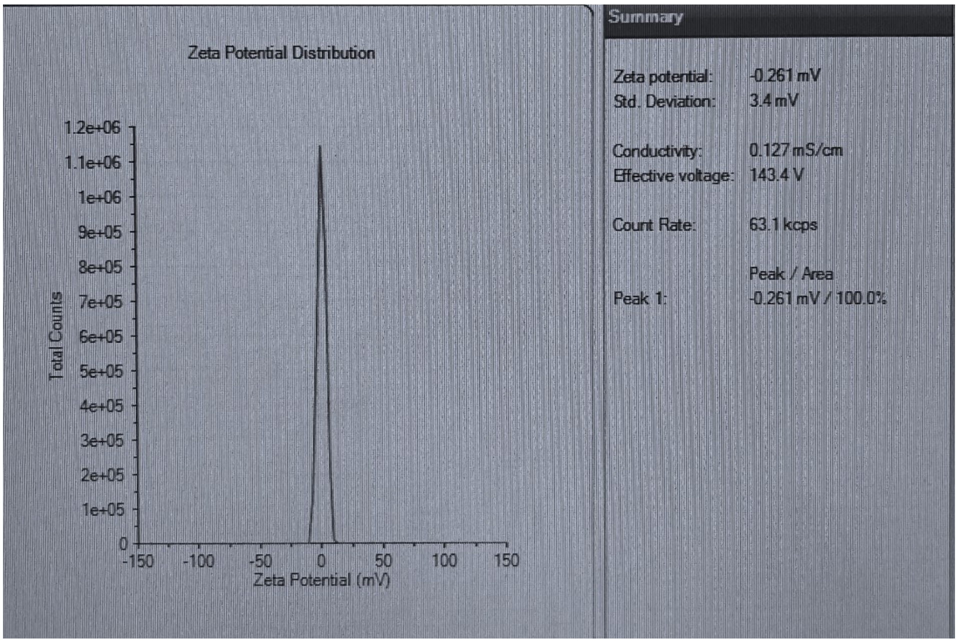

Fig. S3 Gst-**1**-CLArMAs zeta potential.

2.5. Gst-1-CLArMAs pH and temperature stability

The catalytic performance of Gst-**1**-CLArMAs was assessed using harmaline (**2**) as a substrate. Preliminary screening involved varying pH and temperature to establish optimum reaction conditions. The general protocol entailed incubating 450 µL of 2.22 mM harmaline in 0.6 M MES / 3 M HCOONa at pH 6 buffer for 5 min at 45 °C in a shaker. Subsequently, 50 µL of Gst-**1**-CLArMAs suspension was pipetted into the pre-incubated substrate, and shaking at 400 rpm commenced. After 2 h, 50 µL of the

reaction mixture was collected and quenched in 2 mL of 12.5 mM glutathione in a 2:1 MeOH:H_2O solution. The quenched reaction was transferred to a cuvette (1 cm), and UV-vis spectra were recorded to monitor intensity decay at 375 nm, reflecting imine consumption and amine formation. Subsequently, 1 mL of the quenched sample was filtered into an HPLC vial using a 0.22 µm Nylon syringe filter and stored for chiral chromatography analysis.

2.6. Chiral HPLC method for compounds (*R*)-(+)-tetrahydroharmine and (*S*)-(–)-tetrahydroharmine

Enantiomeric excesses were estimated from reverse-phase chiral HPLC traces, acquired according to the following specifications.

Column: PerkinElmer ChromegaChiral CC4, 250 mm × 4.6 mm, 5 µm.

Solvent A: 20 mM $(NH_4)HCO_3$ in HPLC grade H_2O, pH 8.75 adjusted with DEA

Solvent B: Acetonitrile

Injection volume: 20 µL

Mode: isocratic

Solvent ratio: 87.5A:12.5B

Flow: 2 mL min^{-1}

Temperature: 35 °C

Stop time: 20 min

retention times: (*S*)-(–)-tetrahydroharmine 9.4 min and (*R*)-(+)-tetrahydroharmine 10.5 min.

Chiral HPLC: harmaline reduction products

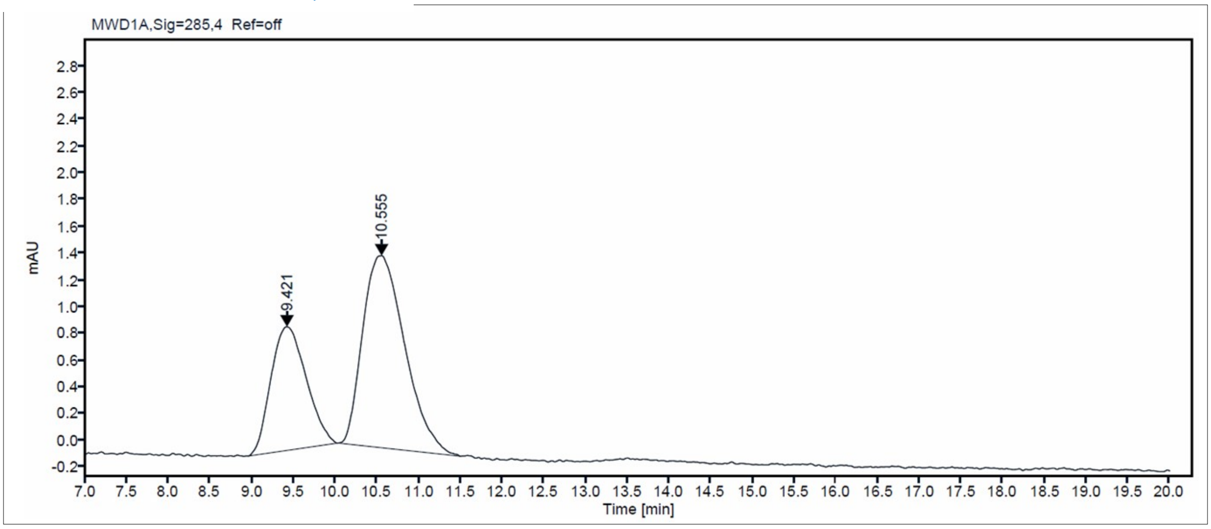

2.7. Recycling studies

The recyclability of CLArMAs under optimised reaction conditions was verified by employing a higher proportion of catalyst and a faster shaking speed to achieve full conversion of harmaline within 1 h in the initial reaction cycle, serving as a control for subsequent cycles. To execute this, 200 µL of a Gst-**1-**CLArMAs suspension was transferred to a 2 mL Eppendorf tube and centrifuged for 5 minutes at 4000

rpm. The supernatant was carefully withdrawn using a micropipette. Subsequently, 500 µL of 2 mM harmaline in 0.6 M MES / 3 M HCOONa at pH 6 buffer was added to the Eppendorf tube that contained the particles and the mixture vigorously vortexed to resuspend the CLArMAs. The Eppendorf tube was promptly transferred to a shaker set to 45 °C and 800 rpm. After 1 h, the tube was centrifuged again for 5 minutes at 4000 rpm, the supernatant was collected, and another reaction cycle commenced in the same manner as the first. This process was repeated for a total of 8 cycles.

2.8. Synthesis of (*S*)-4-cyano-*N*-(1-(pyridin-2-yl)propyl)benzenesulfonamide

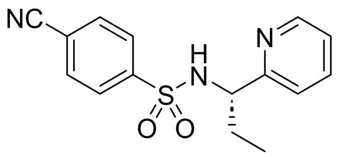

Chemical Formula: $C_{15}H_{15}N_3O_2S$
Molecular Weight: 301.36

(*S*)-1-(Pyridin-2-yl)propan-1-amine (106.5 mg, 0.782 mmol, 1 eq) and triethylamine (0.31 mL; 2.22 mmol, 2.8 eq) were dissolved in anhydrous dichloromethane (8 mL) and placed in an ice bath. A suspension of 4-cyanobenzenesulfonyl chloride (171.8 mg; 0.852 mmol, 1.1 eq) in anhydrous dichloromethane (8 mL) was added dropwise to the reaction mixture. Once the addition was complete, the reaction mixture was allowed to come to room temperature and left stirring for 18 h. The reaction mixture was washed with deionised water (3 x 15 mL). The organic layer was collected and dried over MgSO4, and reduced in vacuo to afford the product as a brown solid (208.4 mg, 0.692 mmol, 88%). The product was used in the next step without further purification.

HRMS (ESI)

Calcd. $[M+H]^+$ ($C_{15}H_{16}N_3O_2S$) m/z = 302.0958; Meas. m/z =302.0961, Mean err (ppm) = 0.1

Calcd. $[M+Na]^+$ ($C_{15}H_{15}N_3NaO_2S$) m/z = 324.0777; Meas. m/z =324.0779, Mean err (ppm) = -2.9

Calcd. $[M+K]^+$ ($C_{15}H_{15}KN_3O_2S$) m/z = 340.0517; Meas. m/z =340.0521, Mean err (ppm) = -0.5

^{1}H NMR (400 MHz, CHLOROFORM-*D*) δ 8.33 (ddd, *J* = 4.9, 1.7, 0.9 Hz, 1H), 7.75 (d, J = 8.5 Hz, 2H), 7.53 (d, J = 8.6 Hz, 2H), 7.48 (td, *J* = 7.7, 1.8 Hz, 1H), 7.07 (ddd, *J* = 7.5, 4.9, 1.1 Hz, 1H), 6.89 (dt, *J* = 7.8, 1.1 Hz, 1H), 6.24 (s, b, 1H), 4.30 (t, *J* = 6.6 Hz, 1H), 1.84 – 1.72 (m, 2H), 0.84 (t, *J* = 7.4 Hz, 3H).

^{13}C NMR (101 MHz, CHLOROFORM-*D*) δ 157.94, 149.14, 144.88, 136.58, 132.40, 127.73, 122.63, 121.76, 117.39, 115.74, 59.44, 30.63, 9.87.

IR ATIR (cm^{-1}) 3290, 3091, 3037, 2966, 2930, 2875, 2234, 1588, 1572, 1432, 1328, 1160, 1087, 1050.

HRMS (ESI): (*S*)-4-cyano-*N*-(1-(pyridin-2-yl)propyl)benzenesulfonamide

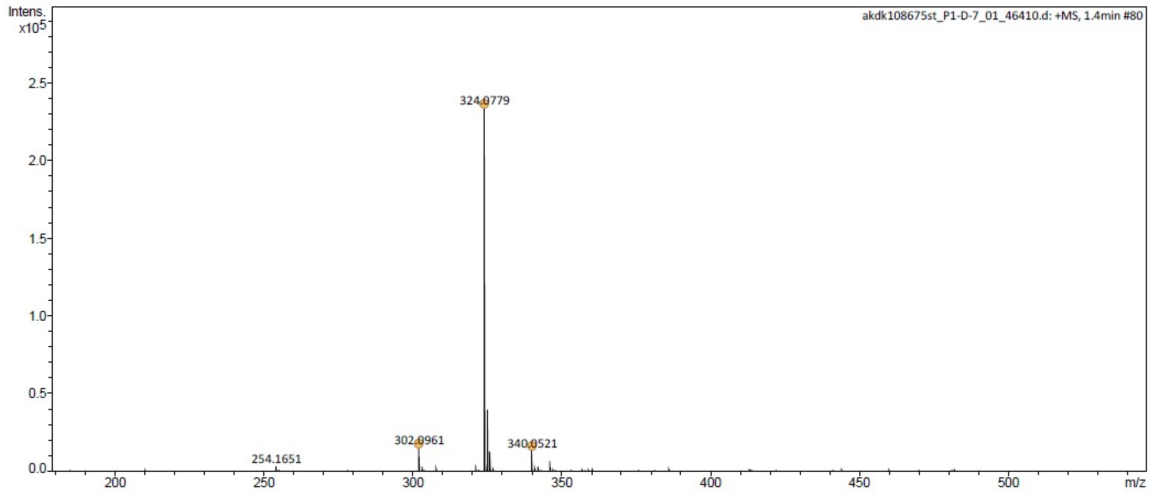

Meas. m/z	#	Ion Formula	m/z	err [ppm]	err [mDa]	mSigma	Mean err [ppm]
302.0961	1	C15H16N3O2S	302.0958	-1.0	-0.3	14.1	0.1
324.0779	1	C15H15N3NaO2S	324.0777	-0.7	-0.2	7.9	-2.9
340.0521	1	C15H15KN3O2S	340.0517	-1.3	-0.4	20.5	-0.5

^{1}H NMR (400 MHz, CHLOROFORM-*D*): (*S*)-4-cyano-*N*-(1-(pyridin-2-yl)propyl)benzenesulfonamide

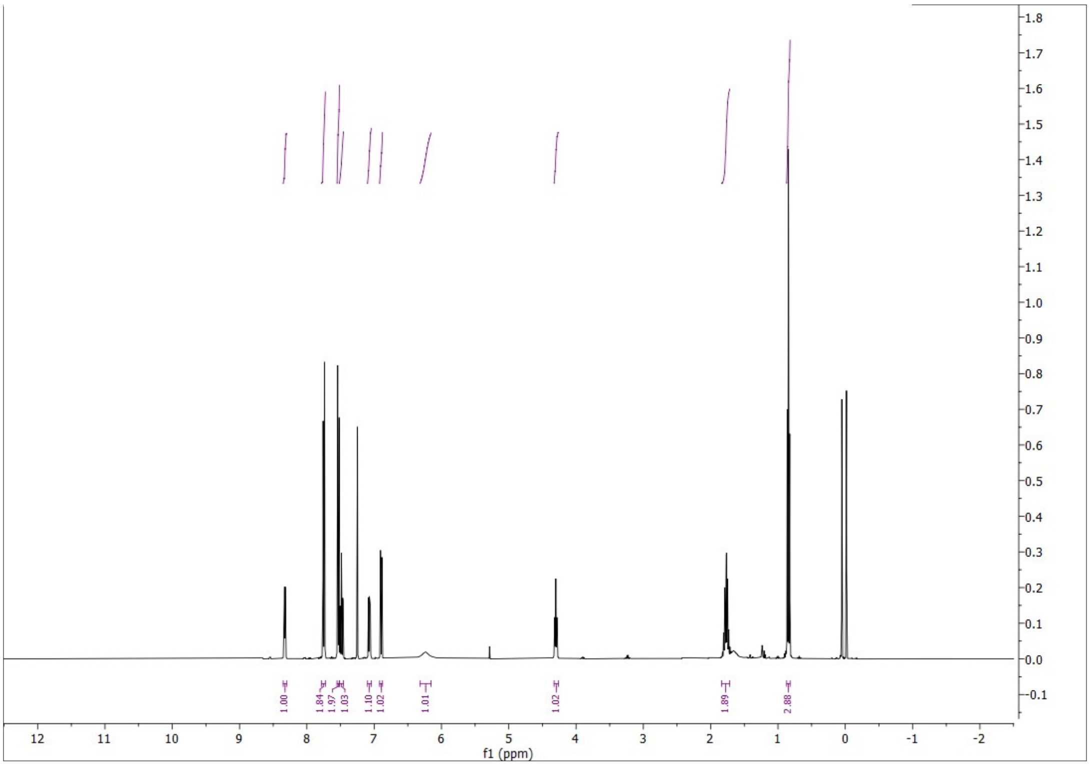

^{13}C NMR (101 MHz, CHLOROFORM-*D*): (*S*)-4-cyano-*N*-(1-(pyridin-2-yl)propyl)benzenesulfonamide

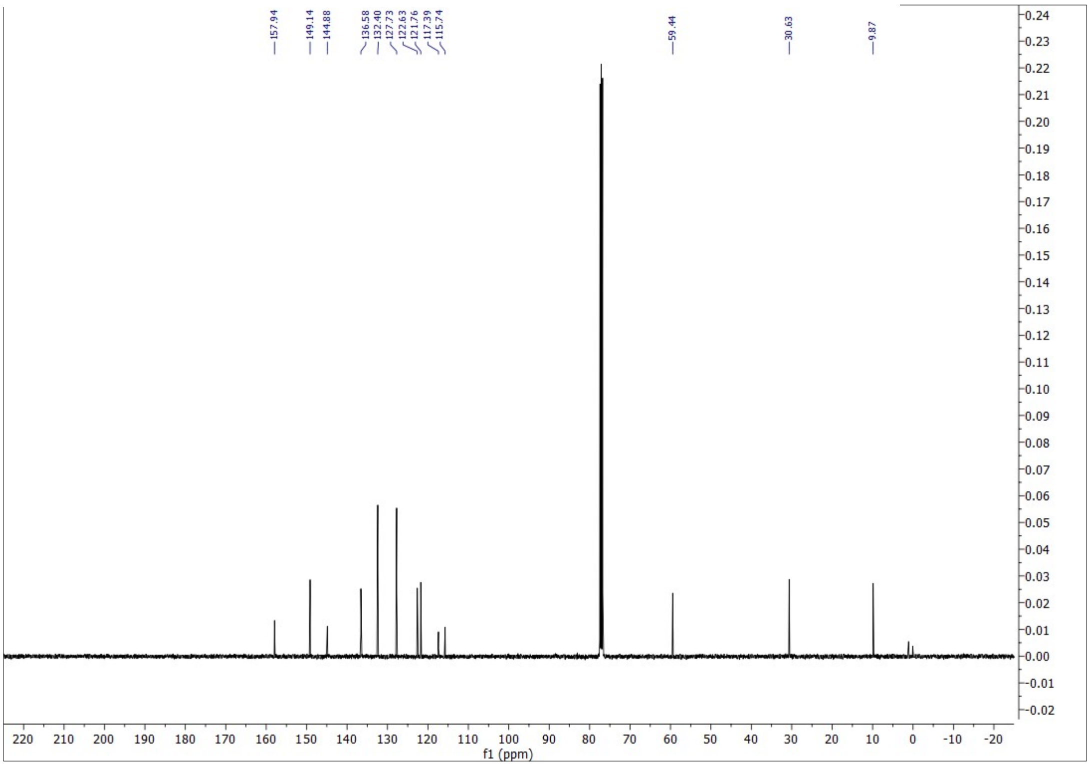

IR ATIR: (*S*)-4-cyano-*N*-(1-(pyridin-2-yl)propyl)benzenesulfonamide

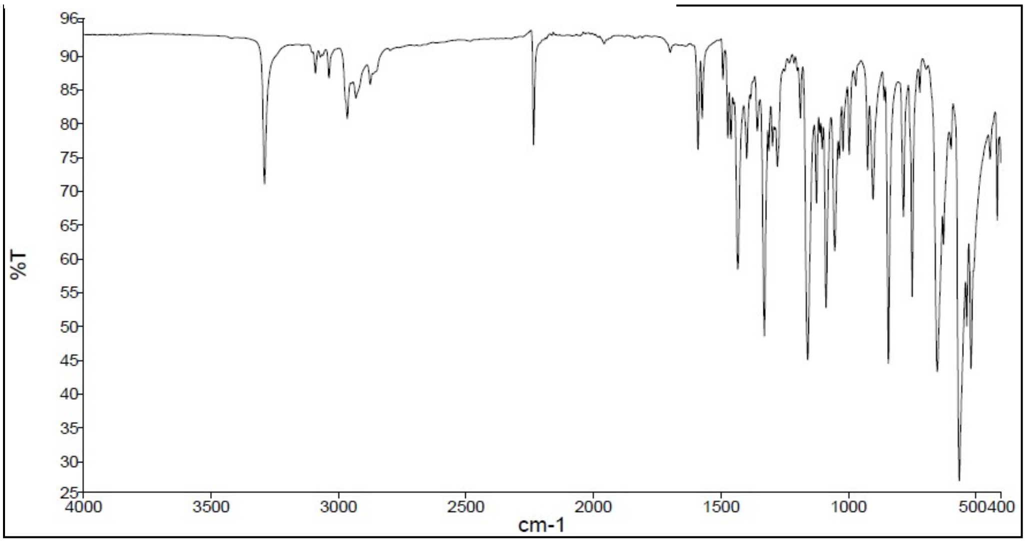

2.9. Synthesis of (*S*)-4-(aminomethyl)-*N*-(1-(pyridin-2-yl)propyl)benzenesulfonamide

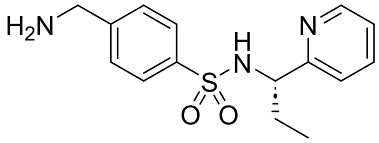

Chemical Formula: $C_{15}H_{19}N_3O_2S$
Molecular Weight: 305.40

(*S*)-4-cyano-N-(1-(pyridin-2-yl)propyl)benzenesulfonamide (198.6 mg, 0.636 mmol) was charged in a flask and filled with approx. 1 atm of H_2. Then Methanol (20 mL), ammonia (7 M MeOH, 400 µL) and Raney Nickel (slurry in water, approx. 400 µL) were added to the flask and kept under H_2. The reaction was stirred for 18 h under a hydrogen atmosphere and then filtered through a pad of celite. The solvent was evaporated in vacuo to afford the desired product as an off-cream/brown solid (188.1 mg, 0.616 mmol, 93%). The crude product was used in the next step without further purification.

HRMS (ESI)

Calcd. $[M+H]^+$ ($C_{15}H_{20}N_3O_2S$) m/z = 306.1271; Meas. m/z = 306.1272, Mean err (ppm) = 0.7

Calcd. $[M+Na]^+$ ($C_{15}H_{19}N_3NaO_2S$) m/z = 328.1090; Meas. m/z = 328.1097, Mean err (ppm) = -1.2

^{1}H NMR (400 MHz, CHLOROFORM-*D*) δ 8.37 – 8.34 (m, 1H), 7.62 (d, J = 8.4 Hz, 2H), 7.43 (td, *J* = 7.7, 1.9 Hz, 1H), 7.20 (d, *J* = 8.0 Hz, 2H), 7.02 (dd, *J* = 7.4, 5.0 Hz, 1H), 6.88 (d, *J* = 7.7 Hz, 1H), 6.12 (s, 1H), 4.26 (q, *J* = 6.8 Hz, 1H), 3.81 (s, 1H, 2H), 1.80 – 1.69 (m, 2H), 0.80 (t, *J* = 7.4 Hz, 3H).

^{13}C NMR (101 MHz, CHLOROFORM-*D*) δ 158.74, 149.04, 149.00, 136.30, 128.17, 127.37, 127.29, 127.18, 122.23, 121.78, 59.21, 45.89, 30.50, 9.82.

IR ATIR (cm^{-1}) 3355, 3287, 3050, 2972, 2960, 2926, 2870, 1590, 1435, 1308, 1152, 1088, 753, 668, 567, 540

HRMS (ESI): (*S*)-4-(aminomethyl)-*N*-(1-(pyridin-2-yl)propyl)benzenesulfonamide

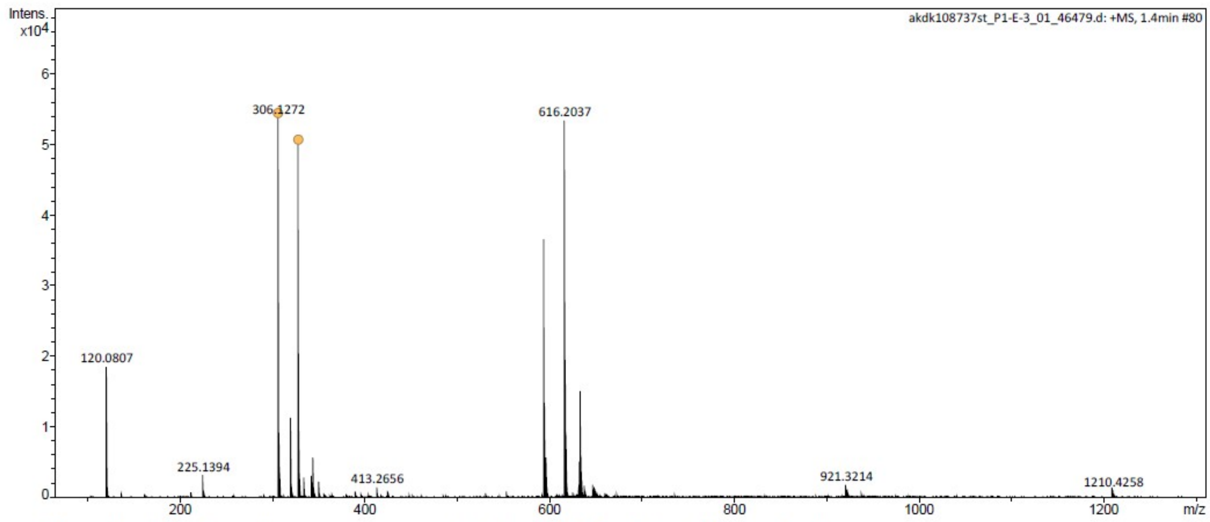

Meas. m/z	#	Ion Formula	m/z	err [ppm]	err [mDa]	mSigma	Mean err [ppm]
306.1272	1	C15H20N3O2S	306.1271	-0.5	-0.2	6.7	0.7
328.1097	1	C15H19N3NaO2S	328.1090	-2.1	-0.7	6.5	-1.2

^{1}H NMR (400 MHz, CHLOROFORM-*D*): (*S*)-4-(aminomethyl)-*N*-(1-(pyridin-2-yl)propyl)benzenesulfonamide

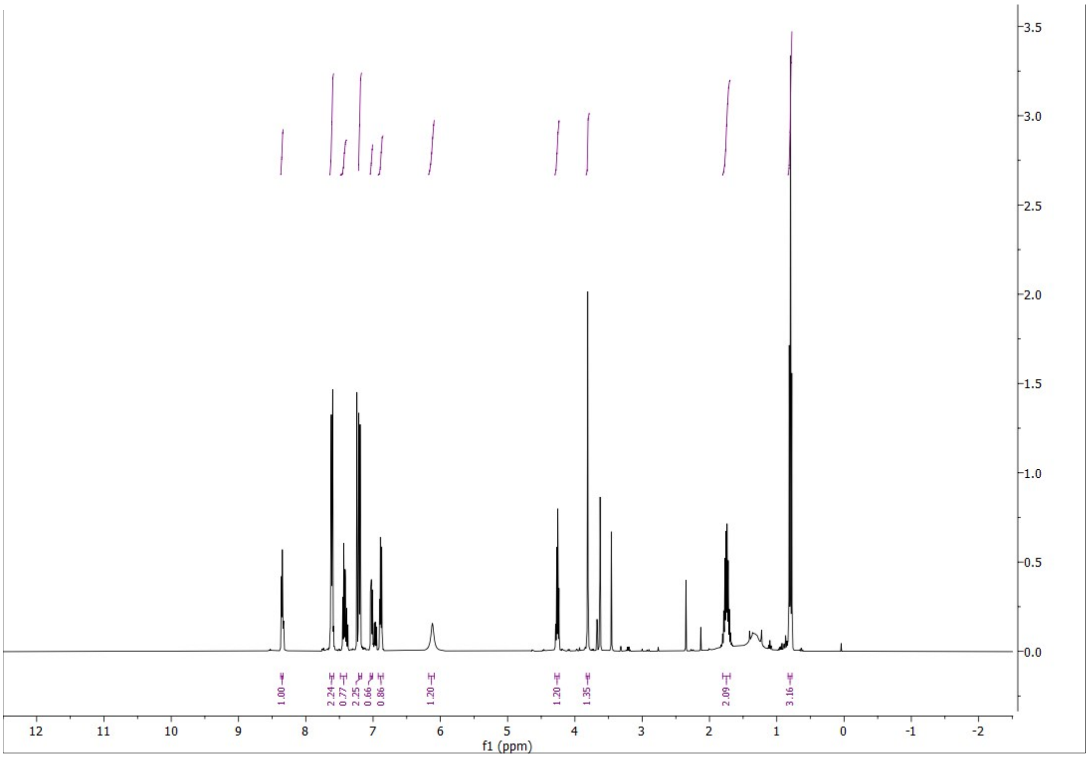

^{13}C NMR (101 MHz, CHLOROFORM-*D*): (*S*)-4-(aminomethyl)-*N*-(1-(pyridin-2-yl)propyl)benzenesulfonamide

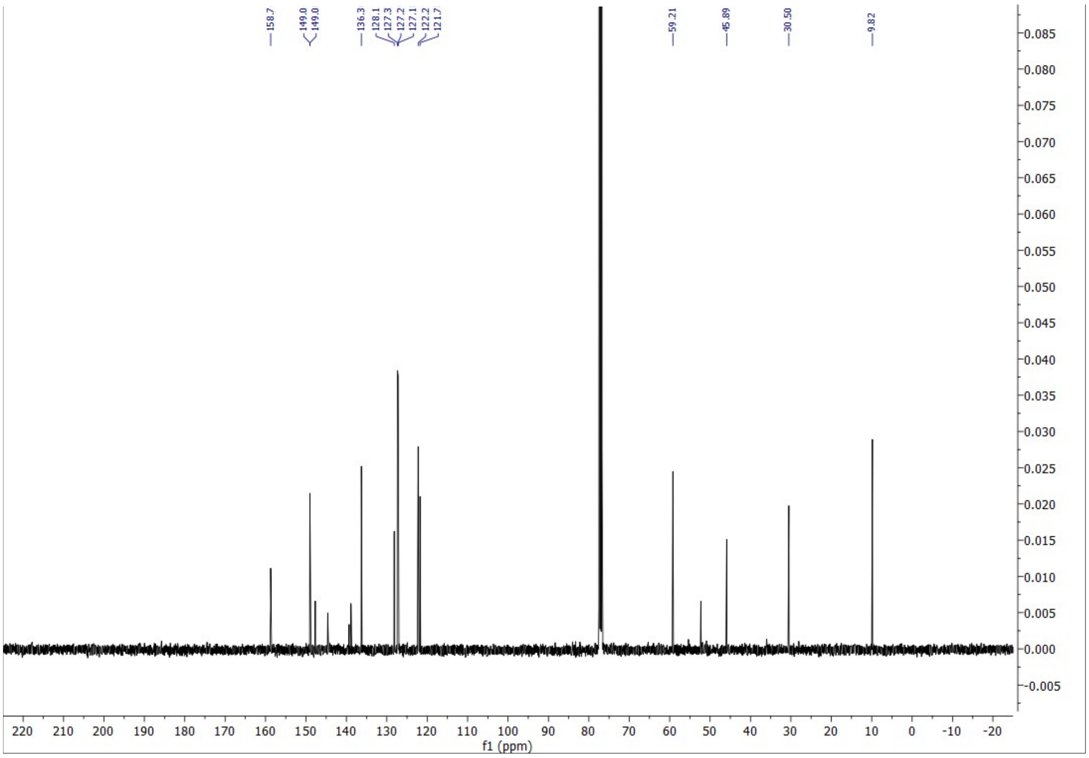

IR ATIR: (*S*)-4-(aminomethyl)-*N*-(1-(pyridin-2-yl)propyl)benzenesulfonamide

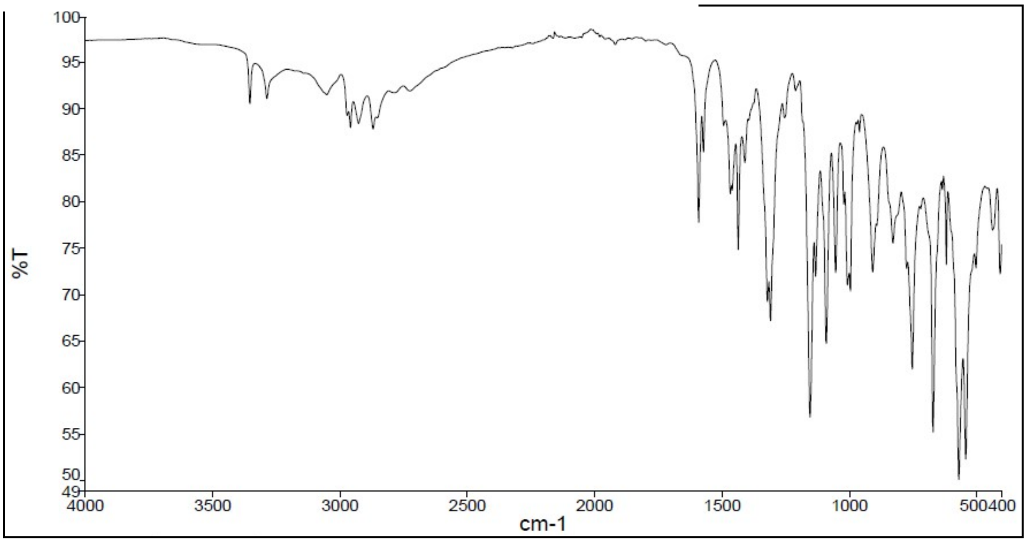

2.10. Synthesis of *N*,*N*'-((*S*)-6-oxo-6-((4-(*N*-((*S*)-1-(pyridin-2-yl)propyl)sulfamoyl)benzyl)amino)hexane-1,5-diyl)bis(2,3-bis(benzyloxy)benzamide)

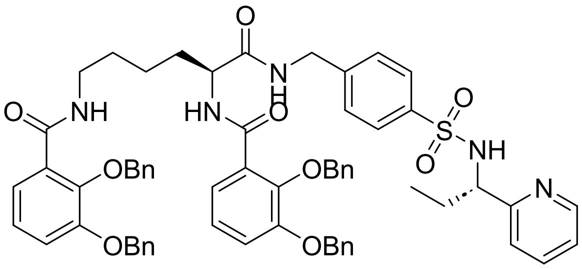

Chemical Formula: $C_{63}H_{63}N_5O_9S$
Molecular Weight: 1066.28

A round bottom flask under an N_2 atmosphere was filled with (Bn_4)-Azotochelin [prepared in accordance with the literature[7]] (444.8 mg, 0.571 mmol, 1.02 eq), 1-Hydroxybenzotriazole monohydrate (85 mg, 0.629 mmol, 1.1 eq), *N*-Ethyl-*N*′-carbodiimide hydrochloride (112.9 mg, 0.589 mmol, 1.1 eq), dry THF (6 mL) and triethylamine (0.16 mL, 1.15 mmol, 2 eq). After 1 h of stirring, a solution of (*S*)-4-(aminomethyl)-*N*-(1-(pyridin-2-yl)propyl)benzenesulfonamide (170.7 mg, 0.559 mmol, 1 eq) in dry THF (6 mL) was added slowly and the mixture was left to react under N_2 at rt for the 18 h.

The reaction mixture was diluted with chloroform (100 mL) and washed with hydrochloric acid (0.1M, 100 mL), saturated sodium hydrogen carbonate (100 mL) and then brine (100 mL). The collected organic layer was dried over $MgSO_4$ and concentrated in vacuo. The crude mixture was purified using column chromatography (AcOEt/MeOH) to afford the product as a white solid (299.3 mg, 0.281 mmol, 49%)

HRMS (ESI)

Calcd. $[M+H]^+$ ($C_{63}H_{64}N_5O_9S$) m/z = 1066.4419; Meas. m/z = 1066.4410, Mean err (ppm) = 1.3

Calcd. $[M+Na]^+$ ($C_{63}H_{63}N_5NaO_9S$) m/z = 1088.4239; Meas. m/z = 1088.4224, Mean err (ppm) = 2.1

Calcd. $[M+K]^+$ ($C_{63}H_{63}KN_5O_9S$) m/z = 1104.3978; Meas. m/z = 1104.3991, Mean err (ppm) = 0.8

^{1}H NMR (400 MHz, CHLOROFORM-*D*) δ 8.35 (d, *J* = 7.2 Hz, 1H), 8.31 (dt, *J* = 5.0, 1.3 Hz, 1H), 7.93 (t, *J* = 5.8 Hz, 1H), 7.64 (ddd, *J* = 9.7, 7.1, 2.5 Hz, 2H), 7.51 (d, *J* = 8.3 Hz, 2H), 7.47 – 7.44 (m, 4H), 7.41 – 7.34 (m, 5H), 7.32 – 7.26 (m, 6H), 7.19 – 7.01 (m, 7H), 6.91 (t, *J* = 6.2 Hz, 1H), 6.81 (d, *J* = 7.7 Hz, 1H), 6.15 (s, 1H), 5.16-5.12 (s, overlapping, 4H), 5.09 (S, 2H), 5.04 (s, 2H), 4.38 (q, J = 6.1 Hz, 1H), 4.29 (d, J = 6.1 Hz, 2H), 4.21 (q, *J* = 6.8 Hz, 1H), 3.16 (q, *J* = 6.7 Hz, 2H), 1.82 – 1.68 (m, 2H), 1.36 – 1.06 (m, 6H), 0.79 (td, *J* = 7.4, 1.3 Hz, 3H).

^{13}C NMR (101 MHz, CHLOROFORM-*D*) δ 171.76, 165.91, 165.20, 158.42, 151.83, 151.77, 148.84, 146.94, 146.82, 143.13, 139.20, 136.47, 136.44, 136.28, 136.16, 128.94, 128.85, 128.82, 128.78, 128.44, 128.37, 127.89, 127.78, 127.37, 127.34, 127.27, 126.41, 124.60, 124.53, 123.31, 123.05, 122.48, 121.91, 117.54, 117.00, 76.47, 76.26, 71.44, 71.35, 59.28, 53.75, 42.61, 39.09, 30.51, 30.37, 28.95, 23.23, 9.91.

IR ATIR (cm^{-1}) 3353, 3278, 3060, 3029, 2927, 2865, 1638, 1573, 1519, 1452, 1310, 1261, 1209, 1156, 1085, 750, 695, 587, 550

HRMS (ESI): *N*,*N*'-((*S*)-6-oxo-6-((4-(*N*-((*S*)-1-(pyridin-2-yl)propyl)sulfamoyl)benzyl)amino)hexane-1,5-diyl)bis(2,3-bis(benzyloxy)benzamide)

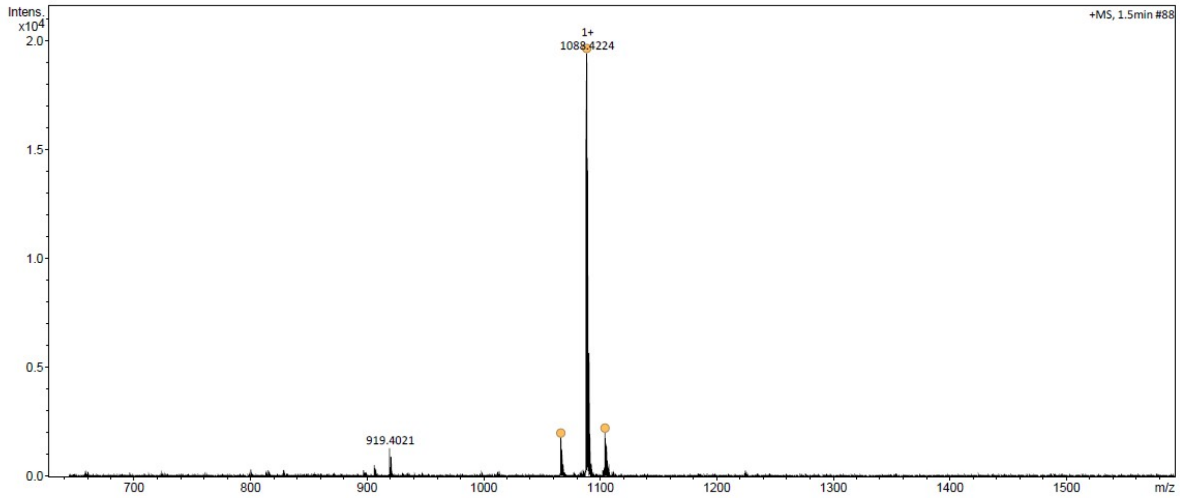

Meas. m/z	#	Ion Formula	m/z	err [ppm]	err [mDa]	mSigma	Mean err [ppm]
1066.440955	1	C63H64N5O9S	1066.441926	0.9	1.0	137.0	1.3
1088.422442	1	C63H63N5NaO9S	1088.423870	1.3	1.4	20.5	2.1
1104.399119	1	C63H63KN5O9S	1104.397808	1.2	1.3	172.9	0.8

^{1}H NMR (400 MHz, CHLOROFORM-*D*): *N*,*N*'-((*S*)-6-oxo-6-((4-(*N*-((*S*)-1-(pyridin-2-yl)propyl)sulfamoyl)benzyl)amino)hexane-1,5-diyl)bis(2,3-bis(benzyloxy)benzamide)

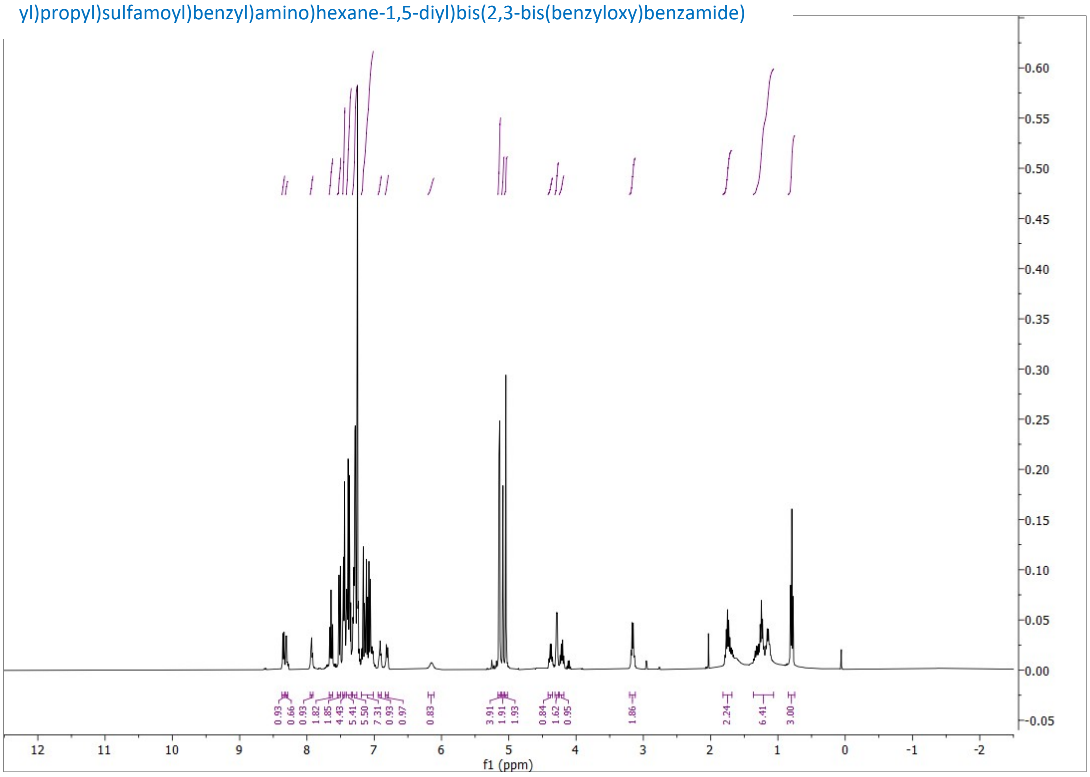

^{13}C NMR (101 MHz, CHLOROFORM-*D*): *N*,*N*'-((*S*)-6-oxo-6-((4-(*N*-((*S*)-1-(pyridin-2-yl)propyl)sulfamoyl)benzyl)amino)hexane-1,5-diyl)bis(2,3-bis(benzyloxy)benzamide)

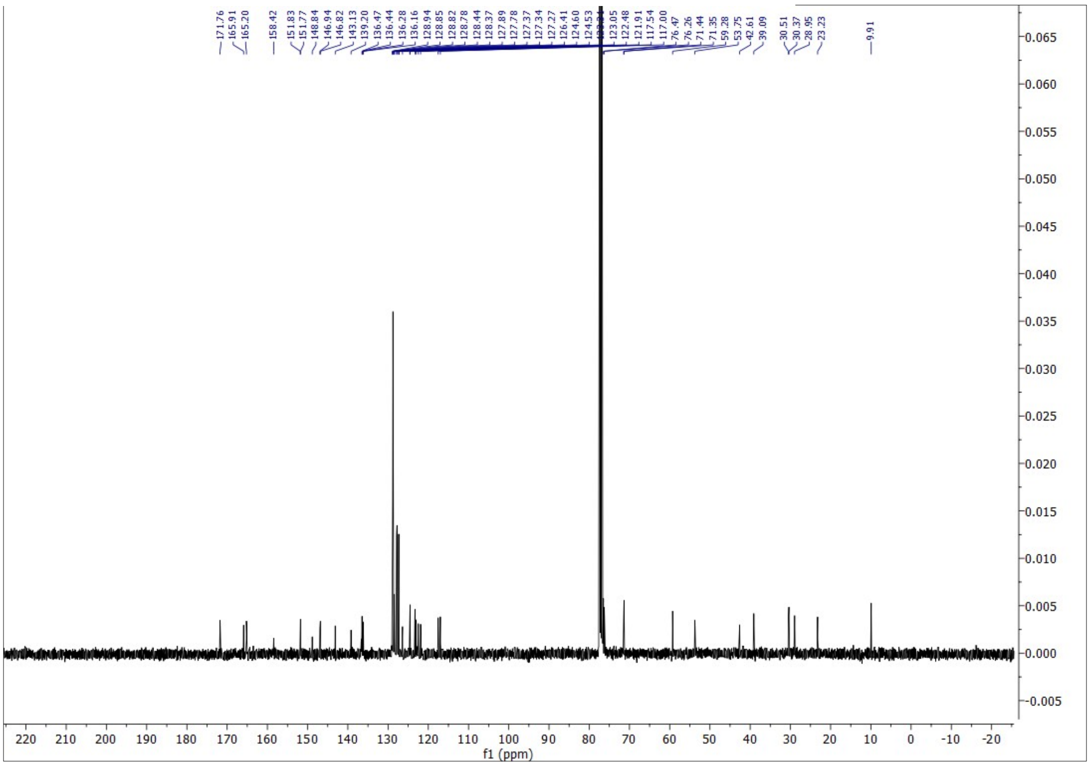

IR ATIR: *N*,*N*'-((*S*)-6-oxo-6-((4-(*N*-((*S*)-1-(pyridin-2-yl)propyl)sulfamoyl)benzyl)amino)hexane-1,5-diyl)bis(2,3-bis(benzyloxy)benzamide)

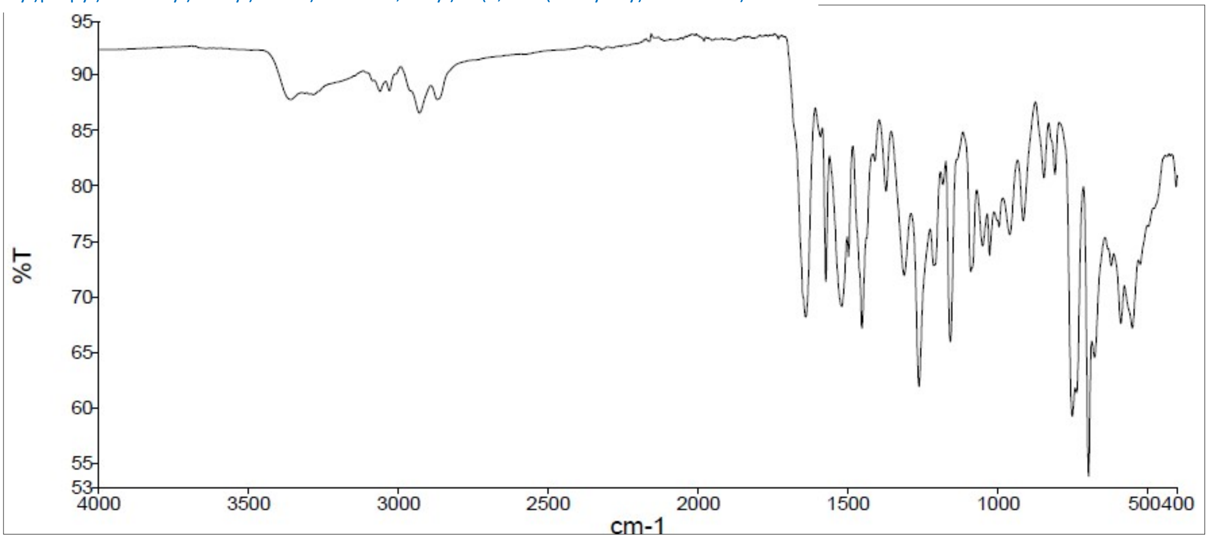

2.11. Synthesis of *N*,*N*'-((*S*)-6-oxo-6-((4-(*N*-((*S*)-1-(pyridin-2-yl)propyl)sulfamoyl)benzyl)amino)hexane-1,5-diyl)bis(2,3-dihydroxybenzamide)

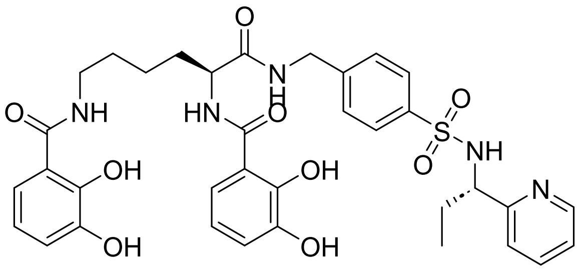

Chemical Formula: $C_{35}H_{39}N_5O_9S$
Molecular Weight: 705.78

All the glassware used in this reaction was previously soaked in HCl (6 M), then successively rinsed with generous amounts of deionised H_2O, concentrated NaOH, deionised H_2O and dry EtOH to make sure it is free from any metal ions and protons.

Dry EtOH (8 mL) and NH_3 (18 M in MeOH, 2 mL) were added to a round bottom flask containing N,N'-((S)-6-oxo-6-((4-(N-((S)-1-(pyridin-2-yl)propyl)sulfamoyl)benzyl)amino)hexane-1,5-diyl)bis(2,3-bis(benzyloxy)benzamide) (101.7 mg, 0.0954 mmol) and palladium hydroxide on carbon (20 wt. % loading, 16.97 mg) under an atmosphere of H_2. The mixture was vigorously stirred for 18 h before being filtered through a Whatman glass microfiber filter GF/F. The product was obtained as a grey/white powder after removing the solvent in vacuo (13 mg, 0.0184 mmol, 19%).

HRMS (ESI):

Calcd. $[M+Na]^+$ ($C_{35}H_{39}N_5NaO_9S$) m/z = 728.2361; Meas. m/z = 728.2371, Mean err (ppm) = -2.7

^{1}H NMR (400 MHz, METHANOL-D_4) δ 8.21 (d, *J* = 5.6 Hz, 1H), 7.52 (dd, *J* = 8.3, 2.5 Hz, 2H), 7.47 (td, *J* = 7.7, 1.8 Hz, 1H), 7.35 – 7.30 (m, 1H), 7.22 (d, *J* = 8.0 Hz, 2H), 7.17 (dd, *J* = 8.1, 1.4 Hz, 1H), 7.10 – 7.04 (m, 2H), 6.89 (dt, *J* = 7.8, 3.8 Hz, 2H), 6.67 (q, *J* = 7.5 Hz, 2H), 4.57 (dd, *J* = 8.7, 5.4 Hz, 1H), 4.34 (td, *J* = 16.0, 9.3 Hz, 2H), 4.22 (td, *J* = 7.3, 2.4 Hz, 1H), 3.38 (t, *J* = 6.9 Hz, 2H), 1.98 – 1.84 (m, 2H), 1.72 – 1.62 (m, 4H), 1.50 (d, *J* = 16.3 Hz, 2H), 0.76 (t, *J* = 7.4 Hz, 3H).

^{13}C NMR (101 MHz, METHANOL-D_4) δ 173.35, 170.20, 169.80, 160.38, 148.06, 146.08, 143.41, 139.76, 137.07, 127.18, 127.16, 126.81, 126.79, 122.34, 121.50, 118.36, 118.12, 117.36, 115.85, 115.53, 60.51, 53.91, 42.02, 38.77, 31.38, 29.34, 28.73, 23.12, 9.54.

IR ATIR (cm^{-1}) 3344, 3059, 2928, 2856, 1637, 1587, 1534, 1455,1320, 1260, 1154, 1090, 740, 675, 586, 550

HRMS (ESI): *N*,*N*'-((*S*)-6-oxo-6-((4-(*N*-((*S*)-1-(pyridin-2-yl)propyl)sulfamoyl)benzyl)amino)hexane-1,5-diyl)bis(2,3-dihydroxybenzamide)

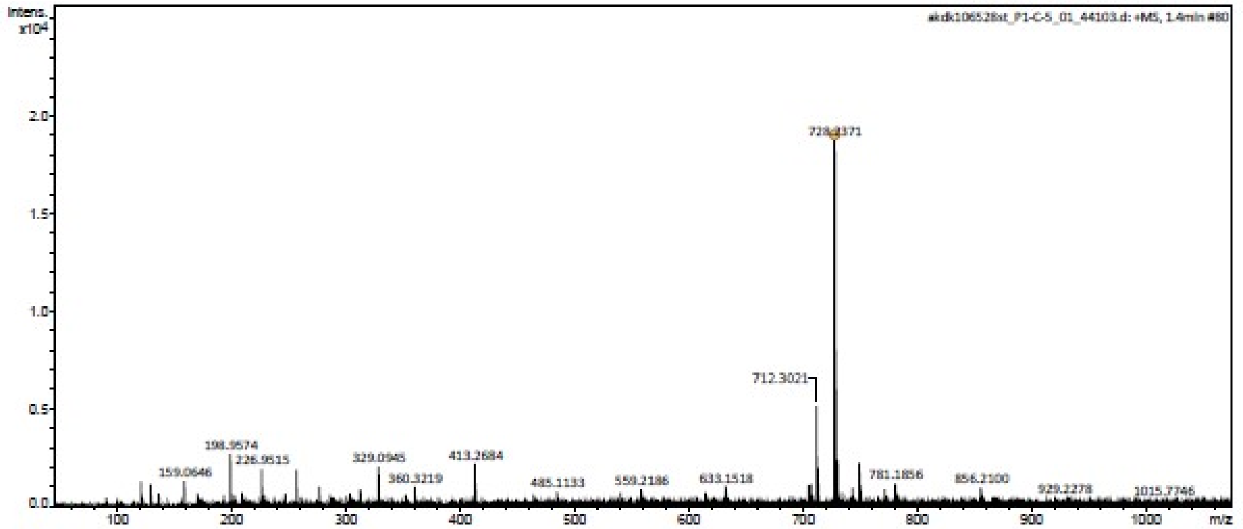

Meas. m/z	#	Ion Formula	m/z	err [ppm]	err [mDa]	mSigma	Mean err [ppm]
728.2371	1	C35H39N5NaO9S	728.2361	-1.4	-1.0	11.4	-2.7

^{1}H NMR (400 MHz,METHANOL-*D$_4$*): *N*,*N*'-((*S*)-6-oxo-6-((4-(*N*-((*S*)-1-(pyridin-2-yl)propyl)sulfamoyl)benzyl)amino)hexane-1,5-diyl)bis(2,3-dihydroxybenzamide)

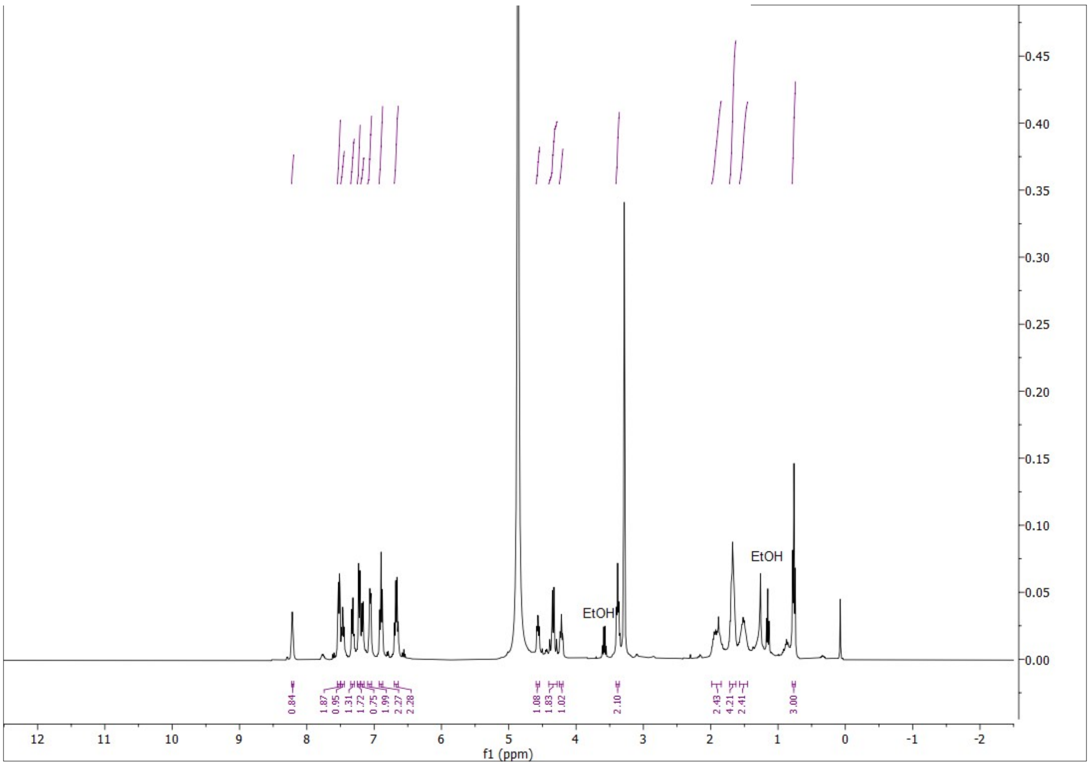

^{13}C NMR (101 MHz, METHANOL-*D$_4$*): *N*,*N*'-((*S*)-6-oxo-6-((4-(*N*-((*S*)-1-(pyridin-2-yl)propyl)sulfamoyl)benzyl)amino)hexane-1,5-diyl)bis(2,3-dihydroxybenzamide)

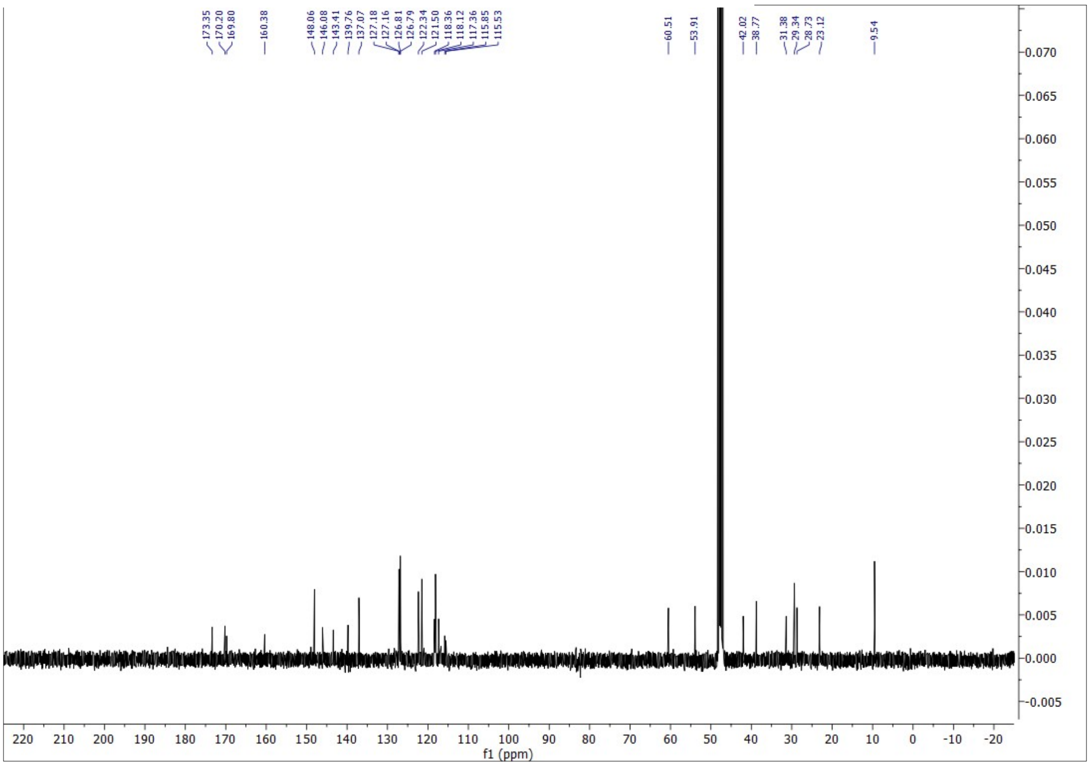

IR ATIR: *N*,*N*'-((*S*)-6-oxo-6-((4-(*N*-((*S*)-1-(pyridin-2-yl)propyl)sulfamoyl)benzyl)amino)hexane-1,5-diyl)bis(2,3-dihydroxybenzamide)

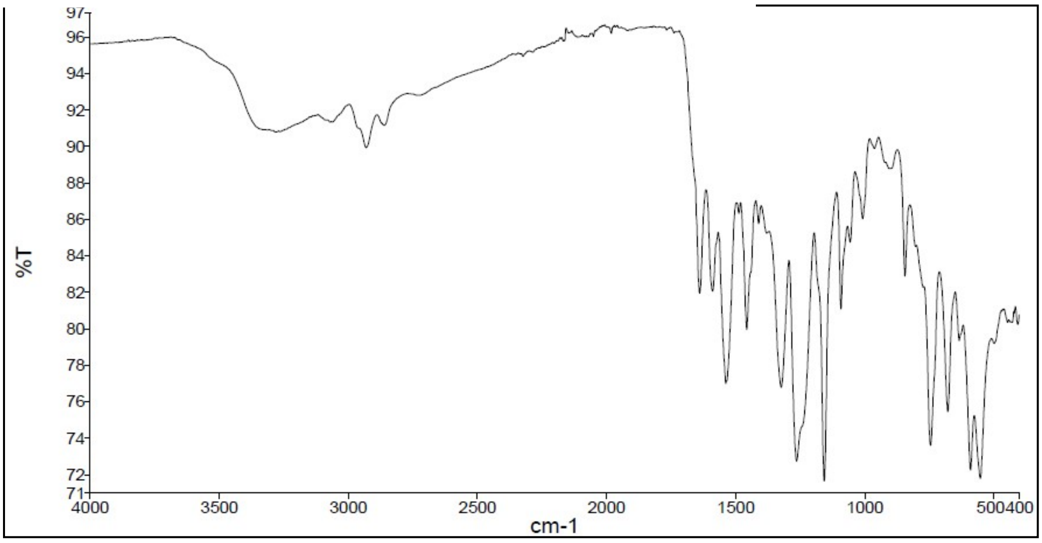

2.12. Synthesis of (*R*)-4-cyano-*N*-(1-(pyridin-2-yl)propyl)benzenesulfonamide

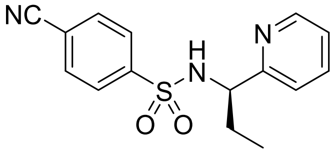

Chemical Formula: $C_{15}H_{15}N_3O_2S$
Molecular Weight: 301.36

(*R*)-1-(Pyridin-2-yl)propan-1-amine dihydrochloride (151.2 mg, 0.723 mmol, 1 eq) and triethylamine (0.64 mL; 4.59 mmol, 6 eq) were dissolved in anhydrous dichloromethane (8 mL) and placed in an ice bath. A suspension of 4-cyanobenzenesulfonyl chloride (167.7 mg; 0.832 mmol, 1.15 eq) in anhydrous dichloromethane (8 mL) was prepared and added dropwise to the reaction mixture. Once the addition was complete, the reaction mixture was allowed to come to room temperature and left stirring for 18 h. The reaction mixture was then washed with deionised water (3 x 15 mL). The organic layer was collected and dried over MgSO4, and reduced in vacuo to afford the product as a cream solid (208.9 mg, 0.680 mmol, 94%). The product was used in the next step without further purification.

HRMS (ESI)

Calcd. $[M+H]^+$ ($C_{15}H_{16}N_3O_2S$) m/z = 302.0963; Meas. m/z = 302.0953, Mean err (ppm) = 1.0

Calcd. $[M+Na]^+$ ($C_{15}H_{15}N_3NaO_2S$) m/z = 324.0783; Meas. m/z = 324.0774, Mean err (ppm) = 1.0

Calcd. $[M+K]^+$ ($C_{15}H_{15}KN_3O_2S$) m/z = 340.0522; Meas. m/z = 340.0507, Mean err (ppm) = 0.8

^{1}H NMR (400 MHz, CHLOROFORM-*D*) δ 8.33 (ddd, *J* = 4.9, 1.8, 1.0 Hz, 1H), 7.75 (dt, *J* = 8.8, 1.8 Hz, 2H), 7.53 (dt, *J* = 8.8, 1.8 Hz, 2H), 7.48 (td, *J* = 7.7, 1.8 Hz, 1H), 7.07 (ddd, *J* = 7.6, 4.8, 1.1 Hz, 1H), 6.89 (dt, *J* = 7.8, 1.0 Hz, 1H), 6.23 (s, b, 1H), 4.30 (t, b, 1H), 1.83 – 1.71 (m, 2H), 0.84 (t, *J* = 7.4 Hz, 3H).

^{13}C NMR (101 MHz, CHLOROFORM-*D*) δ 157.95, 149.16, 144.89, 136.59, 132.42, 127.74, 122.65, 121.77, 117.40, 115.75, 59.45, 30.64, 9.86.

IR ATIR (cm^{-1}) 3290, 3091, 3037, 2966, 2930, 2875, 2234, 1588, 1572, 1432, 1328, 1160, 1087, 1050

HRMS (ESI): (*R*)-4-cyano-*N*-(1-(pyridin-2-yl)propyl)benzenesulfonamide

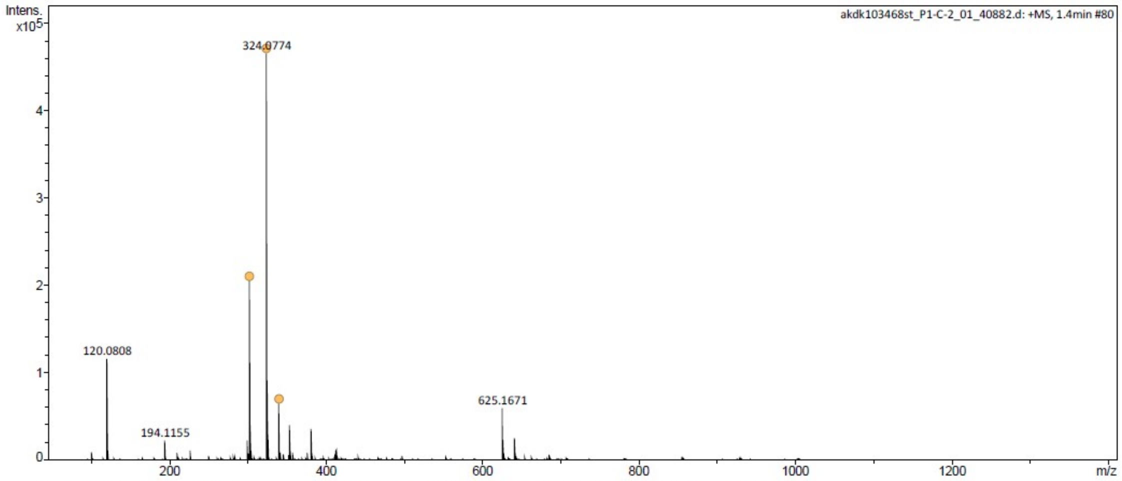

Meas. m/z	#	Ion Formula	m/z	err [ppm]	err [mDa]	mSigma	Mean err [ppm]
302.0953	1	C15H16N3O2S	302.0958	1.7	0.5	5.5	1.0
324.0774	1	C15H15N3NaO2S	324.0777	1.1	0.3	6.1	1.0
340.0507	1	C15H15KN3O2S	340.0517	2.9	1.0	15.1	0.8

^{1}H NMR (400 MHz, CHLOROFORM-*D*): (*R*)-4-cyano-*N*-(1-(pyridin-2-yl)propyl)benzenesulfonamide

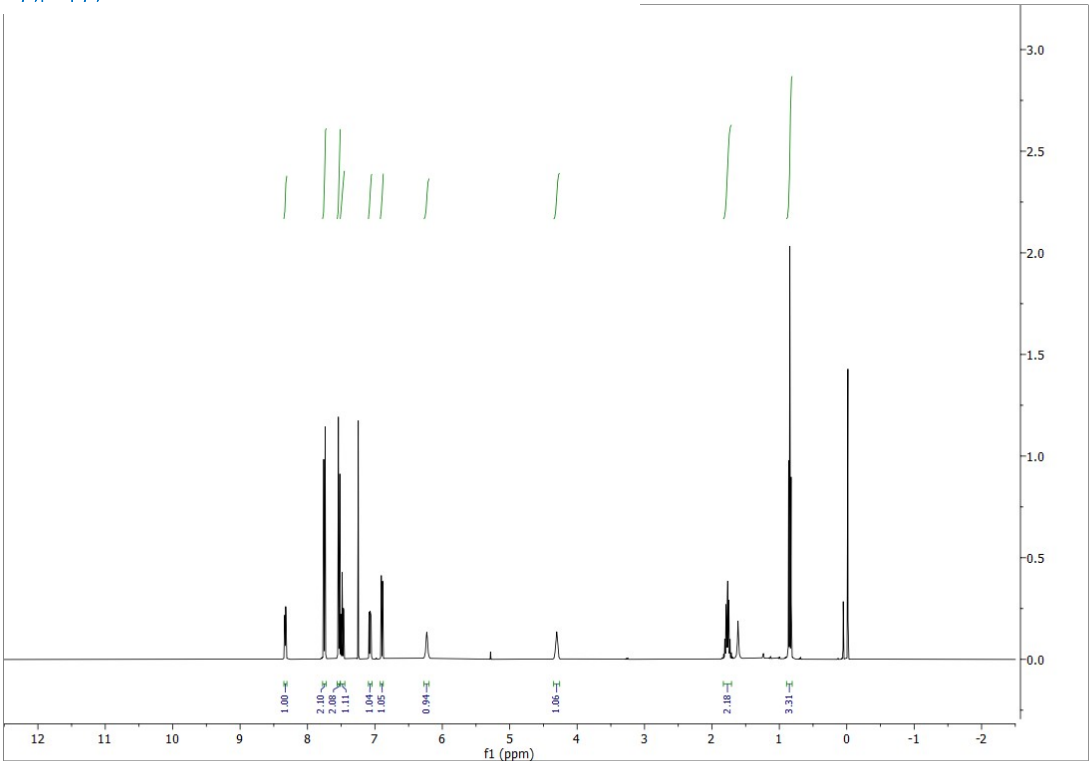

^{13}C NMR (101 MHz, CHLOROFORM-*D*): (*R*)-4-cyano-*N*-(1-(pyridin-2-yl)propyl)benzenesulfonamide

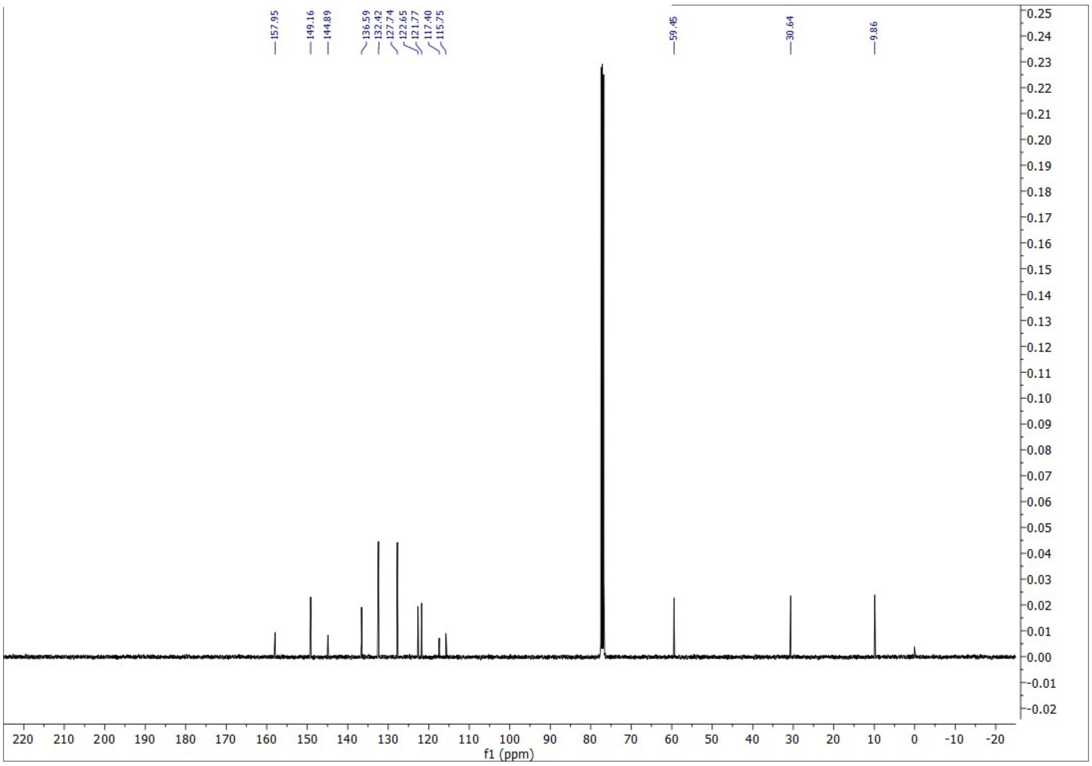

IR ATIR: (*R*)-4-cyano-*N*-(1-(pyridin-2-yl)propyl)benzenesulfonamide

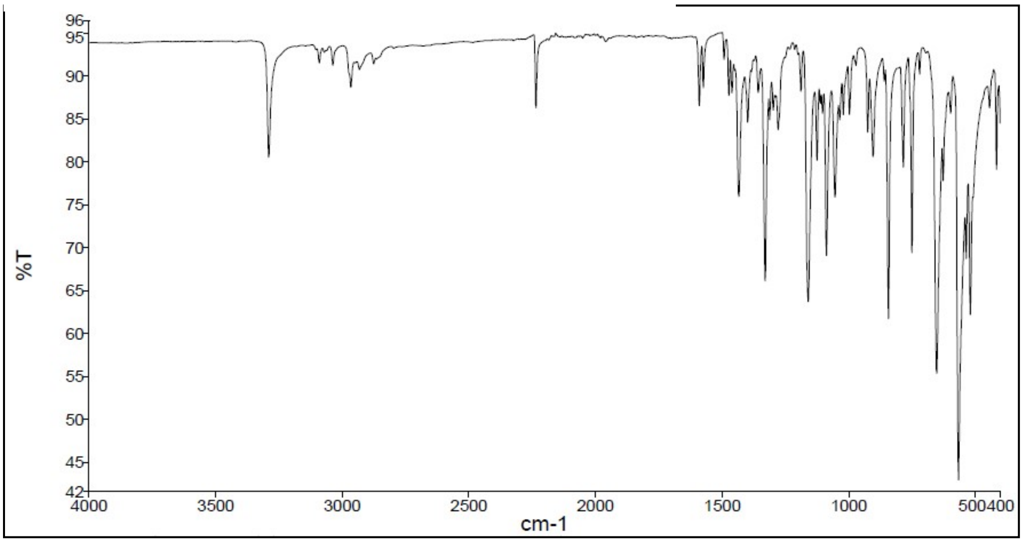

2.13. Synthesis of (*R*)-4-(aminomethyl)-*N*-(1-(pyridin-2-yl)propyl)benzenesulfonamide

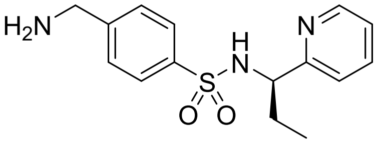

Chemical Formula: $C_{15}H_{19}N_3O_2S$
Molecular Weight: 305.3960

(*R*)-4-cyano-*N*-(1-(pyridin-2-yl)propyl)benzenesulfonamide (208.9 mg, 0.680 mmol) was charged in a flask and filled with approx. 1 atm of H_2. Then Methanol (20 mL), ammonia (7 M MeOH, 400 µL) and Raney Nickel (slurry in water, approx. 400 µL) were added to the flask and kept under H_2. The reaction was stirred for 18 h under a hydrogen atmosphere and then filtered through a pad of celite. The solvent was evaporated in vacuo to afford the desired product as a cream solid (187.9 mg, 0.615 mmol, 90 %). The product was used in the next step without further purification.

HRMS (ESI)

Calcd. $[M+H]^+$ ($C_{15}H_{20}N_3O_2S$) m/z = 306.1271; Meas. m/z = 306.1267, Mean err (ppm) = 1.2

Calcd. $[M+Na]^+$ ($C_{15}H_{19}N_3NaO_2S$) m/z = 328.1090; Meas. m/z = 328.1085, Mean err (ppm) = 1.5

^{1}H NMR (400 MHz, CHLOROFORM-*D*) δ 8.36 (ddd, *J* = 4.9, 1.7, 0.9 Hz, 1H), 7.62 (d, *J* = 8.3 Hz, 2H), 7.44 (td, *J* = 7.7, 1.8 Hz, 1H), 7.21 (d, *J* = 8.1 Hz, 2H), 7.03 (ddd, *J* = 7.6, 4.9, 1.2 Hz, 1H), 6.90 (d, *J* = 8.1 Hz , 1H), 6.12 (s, b, 1H), 4.26 (t, *J* = 6.6 Hz, 1H), 3.83 (s, 2H), 1.84 – 1.69 (m, 2H), 0.80 (t, *J* = 7.4 Hz, 3H).

^{13}C NMR (101 MHz, CHLOROFORM-*D*) δ 158.76, 149.05, 138.91, 136.32, 128.18, 128.01, 127.38, 127.30, 127.22, 122.25, 121.80, 59.22, 45.85, 30.51, 9.83.

IR ATIR (cm^{-1}) 3355, 3287, 3050, 2972, 2960, 2926, 2870, 1590, 1435, 1308, 1152, 1088, 753, 668, 567, 540

HRMS (ESI): (*R*)-4-(aminomethyl)-*N*-(1-(pyridin-2-yl)propyl)benzenesulfonamide

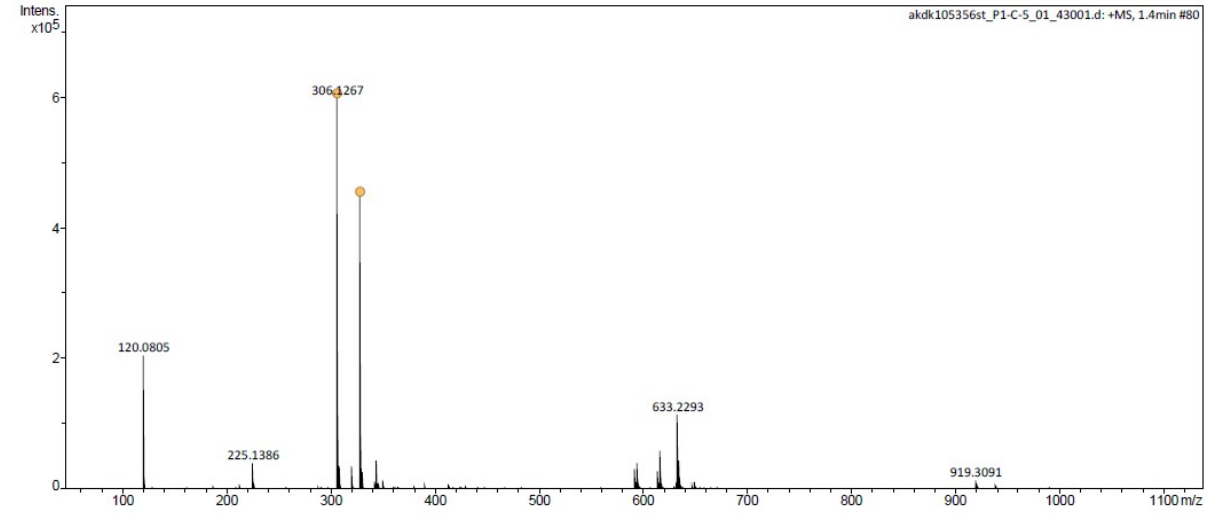

Meas. m/z	#	Ion Formula	m/z	err [ppm]	err [mDa]	mSigma	Mean err [ppm]
306.1267	1	C15H20N3O2S	306.1271	1.4	0.4	4.3	1.2
328.1085	1	C15H19N3NaO2S	328.1090	1.7	0.6	4.0	1.5

^{1}H NMR (400 MHz, CHLOROFORM-*D*): (*R*)-4-(aminomethyl)-*N*-(1-(pyridin-2-yl)propyl)benzenesulfonamide

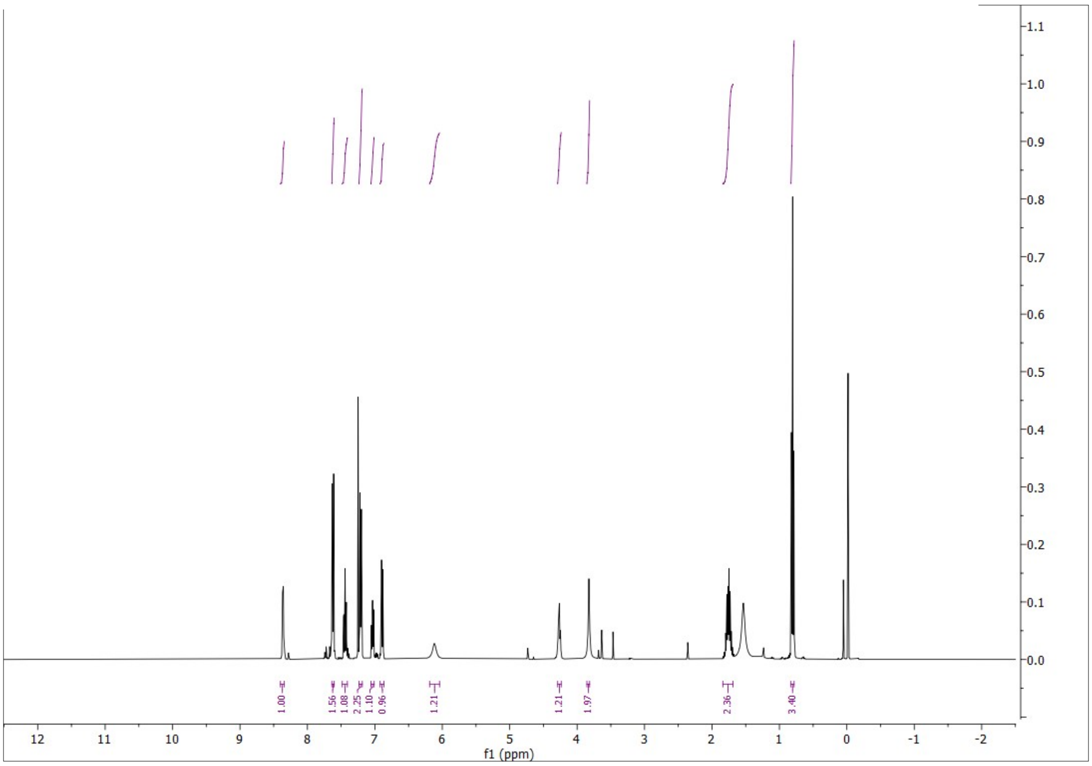

^{13}C NMR (101 MHz, CHLOROFORM-*D*): (*R*)-4-(aminomethyl)-*N*-(1-(pyridin-2-yl)propyl)benzenesulfonamide

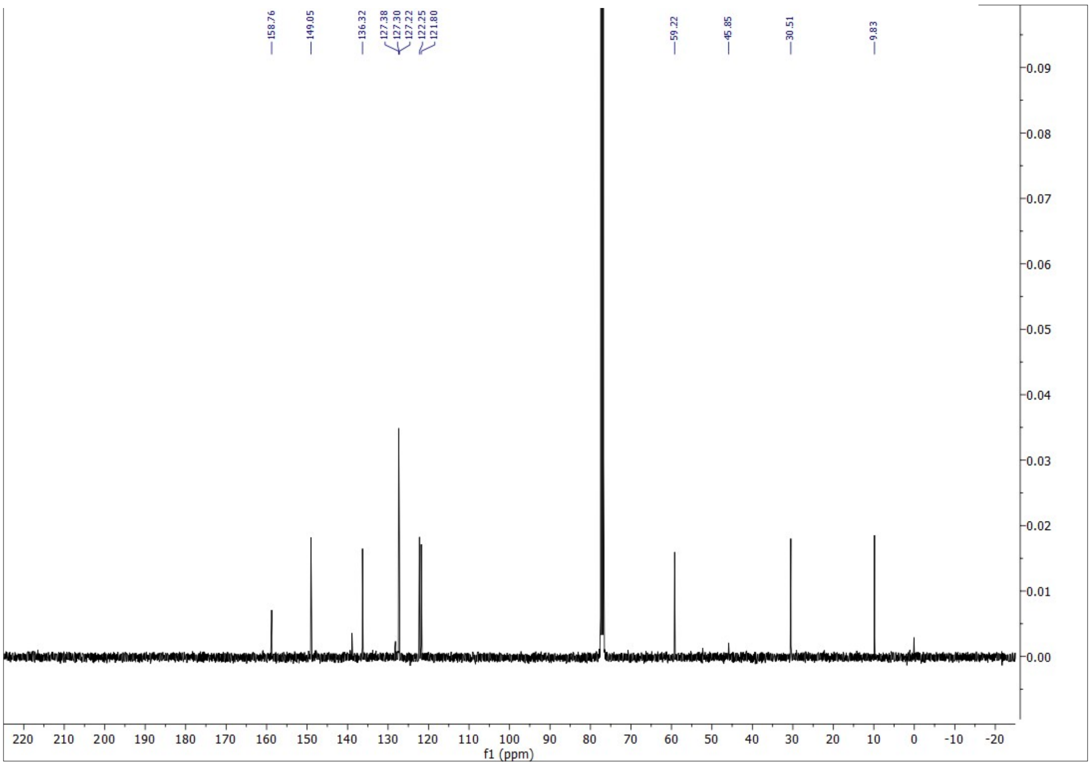

IR ATIR: (*R*)-4-(aminomethyl)-*N*-(1-(pyridin-2-yl)propyl)benzenesulfonamide

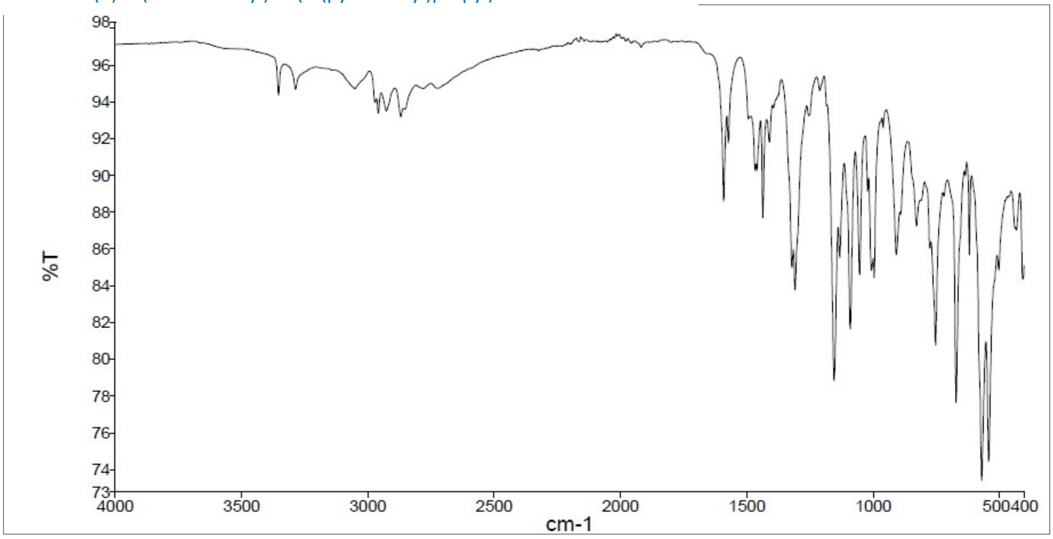

2.14. Synthesis of *N,N*'-((*S*)-6-oxo-6-((4-(*N*-((*R*)-1-(pyridin-2-yl)propyl)sulfamoyl)benzyl)amino)hexane-1,5-diyl)bis(2,3-bis(benzyloxy)benzamide)

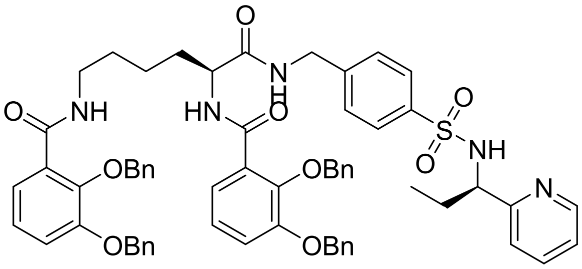

Chemical Formula: $C_{63}H_{63}N_5O_9S$
Molecular Weight: 1066.28

A round bottom flask under an N_2 atmosphere was filled with (Bn_4)-Azotochelin [prepared in accordance with the literature[7]] (446.7 mg, 0.57 mmol, 1 eq), 1-Hydroxybenzotriazole monohydrate (83.1 mg, 0.615 mmol, 1.1 eq), N-Ethyl-N'-carbodiimide hydrochloride (119.3 mg, 0.622 mmol, 1.1 eq), dry THF (6 mL) and triethylamine (0.16 mL, 1.15 mmol, 2 eq). After 1 h of stirring, a solution of (*R*)-4-(aminomethyl)-*N*-(1-(pyridin-2-yl)propyl)benzenesulfonamide (187.9, 0.615 mmol, 1.1 eq) in dry THF (6 mL) was added slowly and the mixture was left to react under N_2 at rt for 48 h.

Then, the reaction mixture was diluted with chloroform (100 mL) and washed with hydrochloric acid solution (0.1M, 100 mL), saturated sodium hydrogen carbonate (100 mL) and then brine (100 mL). The collected organic layer was dried over $MgSO_4$ and concentrated in vacuo. The crude mixture was purified using column chromatography (AcOEt/MeOH) to afford the product as a white solid (375 mg, 0.355 mmol, 62%)

HRMS (ESI):

Calcd. $[M+H]^+$ ($C_{63}H_{64}N_5O_9S$) m/z = 1066.4419; Meas. m/z = 1066.4477, Mean err (ppm) = -3.5

Calcd. $[M+Na]^+$ ($C_{63}H_{63}N_5NaO_9S$) m/z = 1088.4239; Meas. m/z = 1088.4279, Mean err (ppm) = -4.4

Calcd. $[M+K]^+$ ($C_{63}H_{63}KN_5O_9S$) m/z = 1104.3978; Meas. m/z = 1104.4048, Mean err (ppm) = 4.8

^{1}H NMR (400 MHz, CHLOROFORM-*D*) δ 8.35 (d, *J* = 7.2 Hz, 1H), 8.30 (ddt, *J* = 9.7, 4.7, 1.4 Hz, 1H), 7.93 (t, *J* = 5.7 Hz, 1H), 7.64 (ddd, *J* = 8.1, 7.1, 2.4 Hz, 2H), 7.51 (d, J = 8.3 Hz, 2H), 7.47 – 7.43 (m, 4H), 7.41 – 7.34 (m, 7H), 7.34 – 7.26 (m, 6H), 7.20 – 7.06 (m, 6H), 6.99 (dq, J = 4.9, 1.2 Hz, 1H), 6.96 – 6.91 (m, 1H), 6.82 – 6.76 (m, 1H), 6.08 (dd, *J* = 8.5, 2.6 Hz, 1H), 5.14 (s, overlapping, *4*H), 5.08 (s, 2H), 5.04 (s, 2H), 4.38 (q, *J* = 7.1 Hz, 1H), 4.29 (dd, *J* = 6.0, 2.7 Hz, 2H), 4.20 (dd, *J* = 8.4, 6.6 Hz, 1H), 3.16 (q, *J* = 6.7 Hz, 2H), 1.73 (dtd, *J* = 9.8, 7.9, 6.2 Hz, 2H), 1.36 – 1.10 (m, 6H), 0.79 (t, *J* = 1.5 Hz, 3H).

^{13}C NMR (101 MHz, CHLOROFORM-*D*) δ 171.74, 165.95, 165.21, 158.54, 151.84, 151.79, 149.04, 146.96, 146.85, 143.11, 136.49, 136.45, 136.29, 136.15, 128.93, 128.85, 128.83, 128.79, 128.46, 128.38, 127.89, 127.78, 127.37, 127.28, 126.40, 124.60, 124.54, 123.34, 123.09, 122.37, 121.84, 117.57, 117.02, 77.31, 76.48, 76.28, 71.46, 71.36, 59.28, 53.76, 42.61, 39.08, 30.42, 28.94, 23.22, 9.87.

IR ATIR (cm^{-1}) 3360, 3062, 3030, 2929, 2868, 1680, 1574, 1520, 1452, 1260, 1157, 1087, 750, 696

HRMS (ESI): *N*,*N*'-((*S*)-6-oxo-6-((4-(*N*-((*R*)-1-(pyridin-2-yl)propyl)sulfamoyl)benzyl)amino)hexane-1,5-diyl)bis(2,3-bis(benzyloxy)benzamide)

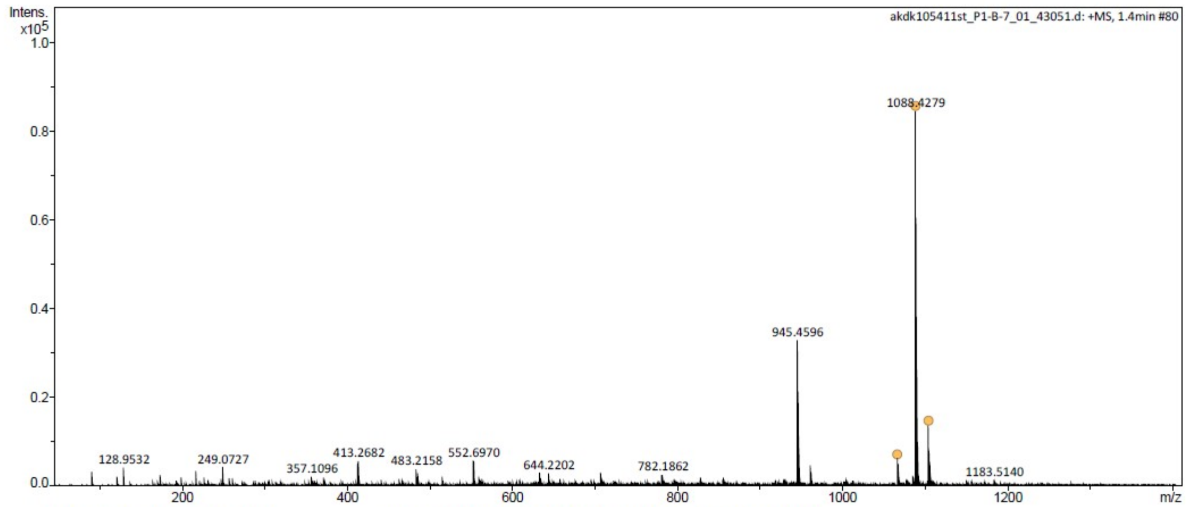

Meas. m/z	#	Ion Formula	m/z	err [ppm]	err [mDa]	mSigma	Mean err [ppm]
1066.4477	1	C63H64N5O9S	1066.4419	-5.4	-5.7	56.1	-3.5
1088.4279	1	C63H63N5NaO9S	1088.4239	-3.7	-4.0	7.2	-4.4
1104.4048	1	C63H63KN5O9S	1104.3978	-6.3	-7.0	56.3	-4.8

^{1}H NMR (400 MHz, CHLOROFORM-*D*): *N*,*N*'-((*S*)-6-oxo-6-((4-(*N*-((*R*)-1-(pyridin-2-yl)propyl)sulfamoyl)benzyl)amino)hexane-1,5-diyl)bis(2,3-bis(benzyloxy)benzamide)

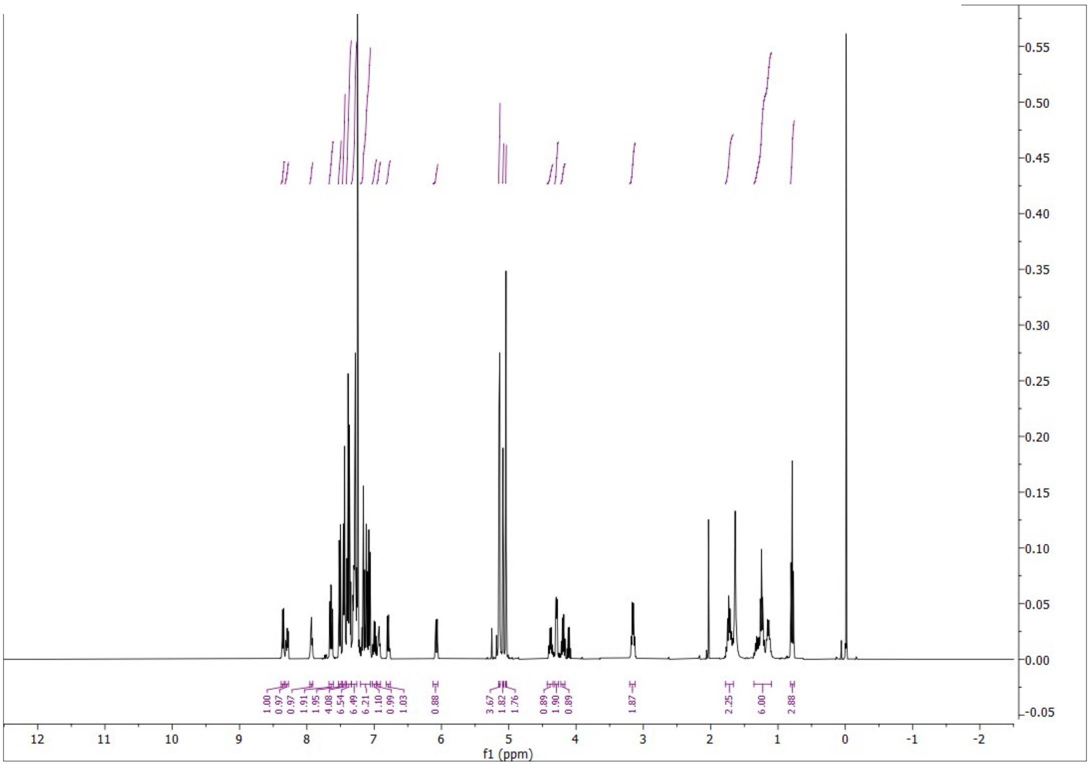

^{13}C NMR (101 MHz, CHLOROFORM-*D*): *N*,*N*'-((*S*)-6-oxo-6-((4-(*N*-((*R*)-1-(pyridin-2-yl)propyl)sulfamoyl)benzyl)amino)hexane-1,5-diyl)bis(2,3-bis(benzyloxy)benzamide)

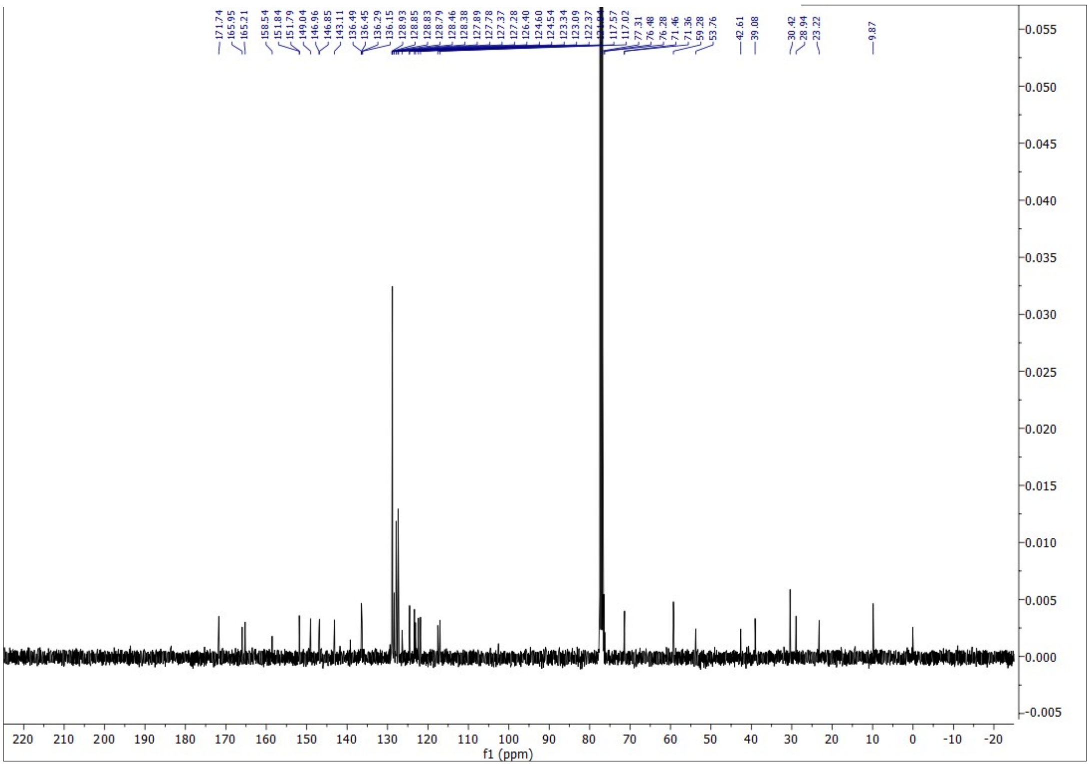

IR ATIR: *N*,*N*'-((*S*)-6-oxo-6-((4-(*N*-((*R*)-1-(pyridin-2-yl)propyl)sulfamoyl)benzyl)amino)hexane-1,5-diyl)bis(2,3-bis(benzyloxy)benzamide)

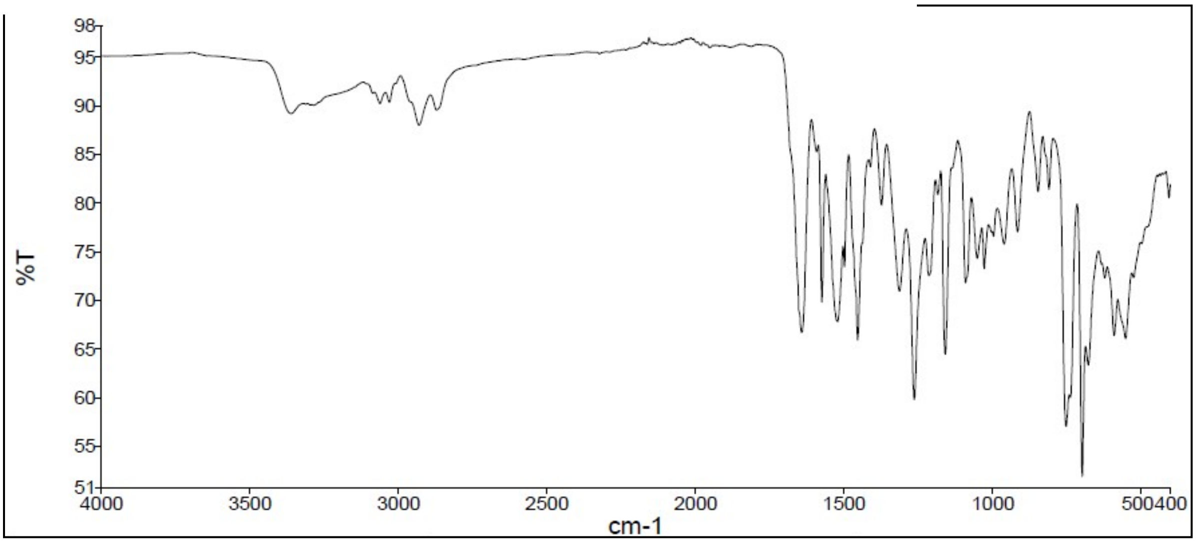

2.15. Synthesis of *N*,*N*'-((*S*)-6-oxo-6-((4-(*N*-((*R*)-1-(pyridin-2-yl)propyl)sulfamoyl)benzyl)amino)hexane-1,5-diyl)bis(2,3-dihydroxybenzamide)

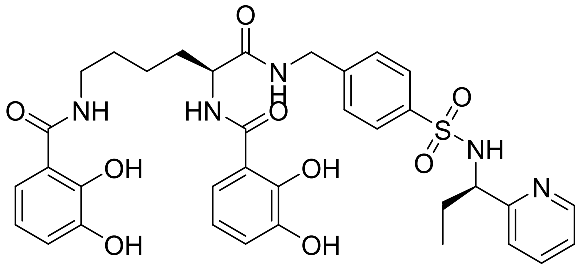

Chemical Formula: $C_{35}H_{39}N_5O_9S$
Molecular Weight: 705.78

All the glassware used in this reaction was previously soaked in HCl (6 M), then successively rinsed with generous amount of deionised H_2O, concentrated NaOH, deionised H_2O and then dry EtOH to make sure it is free from any metal ions and protons.

Dry EtOH (8 mL) and NH_3 (18 M in MeOH, 2 mL) were added to a round bottom flask containing *N,N*'-((*S*)-6-oxo-6-((4-(N-((*R*)-1-(pyridin-2-yl)propyl)sulfamoyl)benzyl)amino)hexane-1,5-diyl)bis(2,3-bis(benzyloxy)benzamide) (113.6 mg, 0.107 mmol) and palladium hydroxide on carbon (20 wt. % loading, 16.94 mg) under an atmosphere of H_2. The mixture was vigorously stirred for 18 h before being filtered through a Whatman glass microfiber filter GF/F. The product was obtained as a grey/white powder after removing the solvent in vacuo (30.5 mg, 0.0432 mmol, 40%).

HRMS (ESI):

Calcd. $[M+H]^+$ ($C_{35}H_{40}N_5O_9S$) m/z = 706.2541; Meas. m/z = 706.2558, Mean err (ppm) = -1.2

Calcd. [M+Na]+ ($C_{35}H_{39}N_5NaO_9S$) m/z = 728.2361; Meas. m/z = 728.2373, Mean err (ppm) = -2.2

Calcd. [M+K]+ ($C_{35}H_{38}N_5Na_2O_9S$) m/z = 750.2180; Meas. m/z = 750.2180, Mean err (ppm) = 1.0

^{1}H NMR (400 MHz, METHANOL-*D*$_4$) δ 8.21 (t, *J* = 1.2 Hz, 1H), 7.52 (dd, *J* = 8.3, 2.3 Hz, 2H), 7.46 (t, *J* = 7.8 Hz, 1H), 7.32 (d, *J* = 8.1 Hz, 1H), 7.22 (d, *J* = 8.0 Hz, 2H), 7.17 (d, *J* = 8.1 Hz, 1H), 7.05 (t, *J* = 5.4 Hz, 2H), 6.90 (t, *J* = 8.7 Hz, 2H), 6.68 (dt, *J* = 11.0, 7.9 Hz, 2H), 4.57 (t, *J* = 5.8 Hz, 1H), 4.34 (q, *J* = 15.8 Hz, 2H), 4.21 (t, *J* = 7.2 Hz, 1H), 3.37 (t, *J* = 6.9 Hz, 2H), 3.28 (t, *J* = 1.7 Hz, 1H), 2.00 – 1.82 (m, 2H), 1.72 – 1.62 (m, 4H), 1.58 – 1.43 (m, 2H), 0.75 (t, *J* = 7.3 Hz, 3H).

^{13}C NMR (101 MHz, METHANOL-*D*$_4$) δ 173.32, 170.22, 169.80, 160.38, 148.96, 148.54, 148.08, 146.03, 145.97, 143.42, 139.75, 137.08, 127.20, 126.83, 122.36, 121.53, 118.44, 118.32, 118.25, 117.34, 115.82, 115.51, 60.53, 60.51, 53.92, 42.05, 38.80, 31.37, 29.34, 28.73, 23.12, 9.54.

IR ATIR (cm^{-1}) 3342, 3067, 2930, 2961, 1636, 1586, 1631, 1455, 1320, 1260, 1154, 743, 675, 587, 550

HRMS (ESI): *N*,*N*'-((*S*)-6-oxo-6-((4-(*N*-((*R*)-1-(pyridin-2-yl)propyl)sulfamoyl)benzyl)amino)hexane-1,5-diyl)bis(2,3-dihydroxybenzamide)

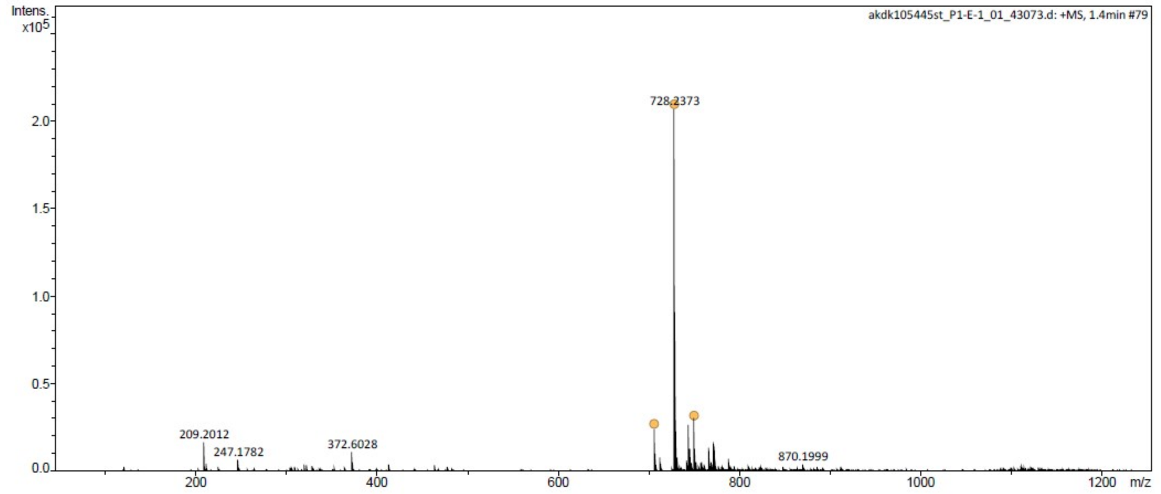

Meas. m/z	#	Ion Formula	m/z	err [ppm]	err [mDa]	mSigma	Mean err [ppm]
706.2558	1	C35H40N5O9S	706.2541	-2.4	-1.7	9.9	-1.2
728.2373	1	C35H39N5NaO9S	728.2361	-1.8	-1.3	12.0	-2.2
750.2180	1	C35H38N5Na2O9S	750.2180	-0.0	-0.0	17.2	1.0

^{1}H NMR (400 MHz,METHANOL-*D$_4$*): *N*,*N*'-((*S*)-6-oxo-6-((4-(*N*-((*R*)-1-(pyridin-2-yl)propyl)sulfamoyl)benzyl)amino)hexane-1,5-diyl)bis(2,3-dihydroxybenzamide)

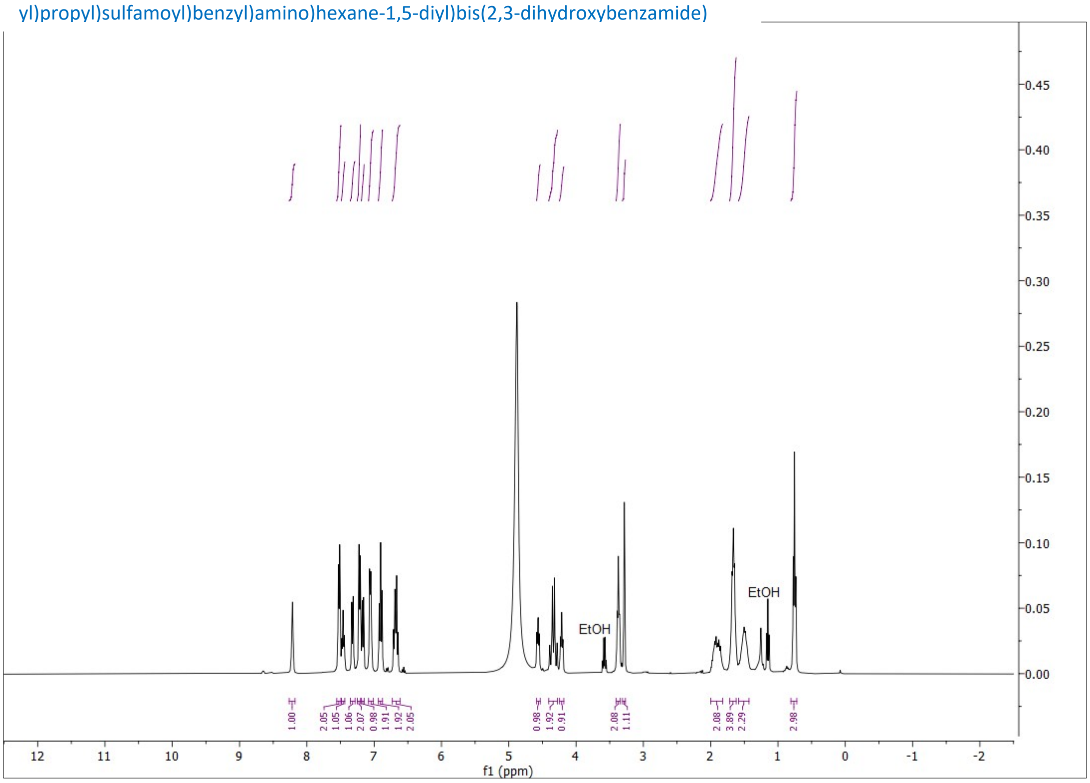

^{13}C NMR (101 MHz, METHANOL-*D$_4$*): *N*,*N*'-((*S*)-6-oxo-6-((4-(*N*-((*R*)-1-(pyridin-2-yl)propyl)sulfamoyl)benzyl)amino)hexane-1,5-diyl)bis(2,3-dihydroxybenzamide)

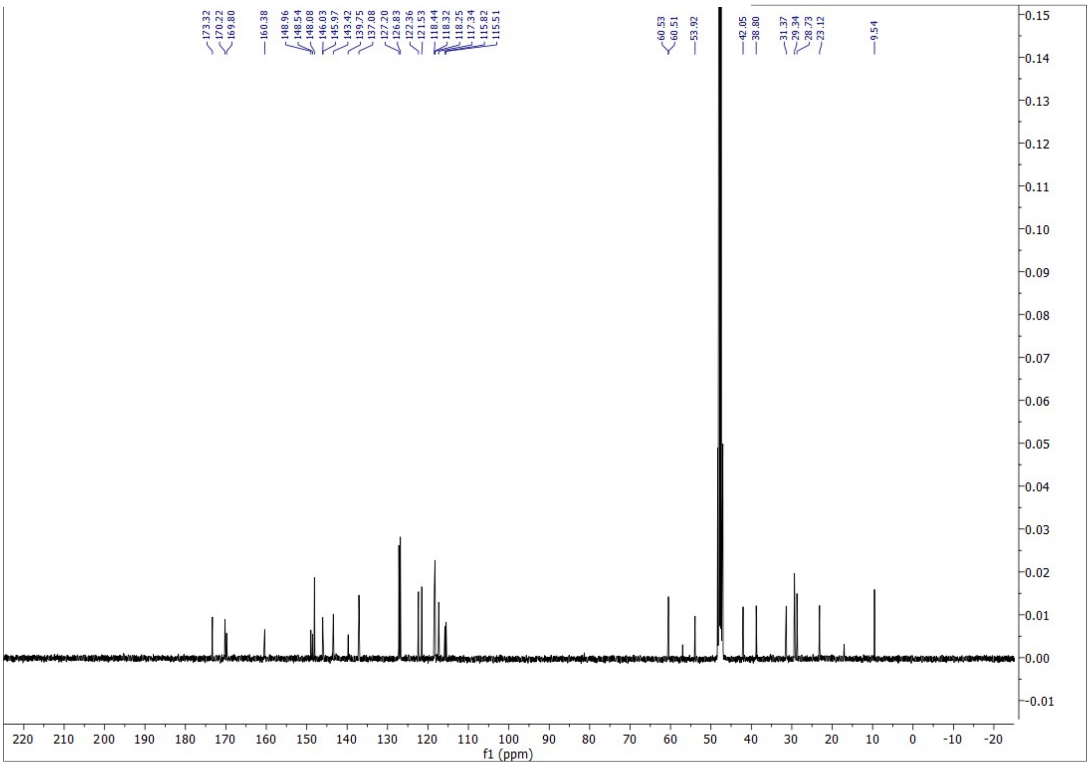

IR ATIR: *N*,*N*'-((*S*)-6-oxo-6-((4-(*N*-((*R*)-1-(pyridin-2-yl)propyl)sulfamoyl)benzyl)amino)hexane-1,5-diyl)bis(2,3-dihydroxybenzamide)

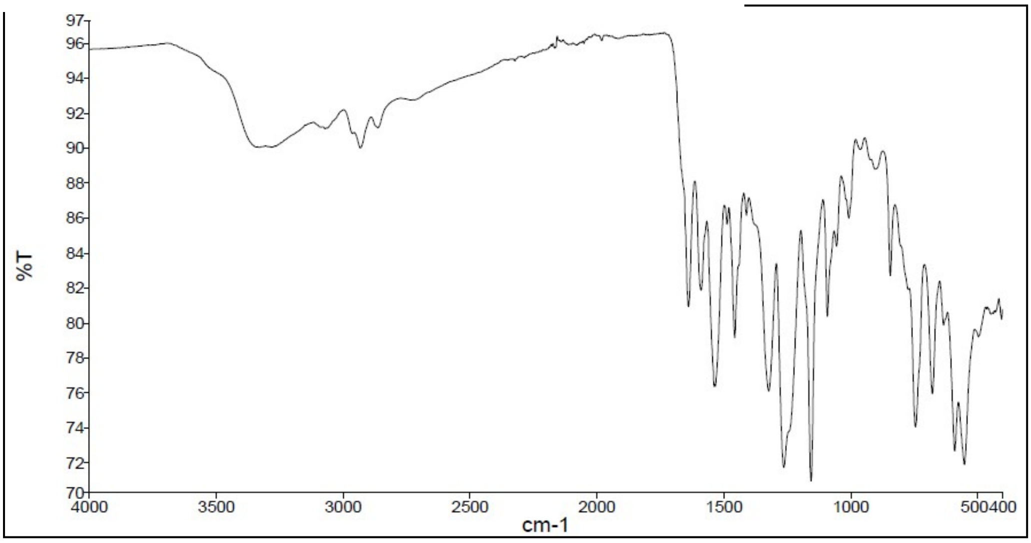

2.16. CD-spectroscopic investigation of 1, 4, 5, Gst-1, Gst-4, Gst-5

Samples were prepared in a 1 cm quartz cuvette. CD spectra were recorded in continuous mode with a range of 240-700 nm, 0.5 nm pitch, 100 nm/min scanning speed, 2 s response, 2 nm bandwidth, and 3 accumulations. A blank buffer sample of 100 mM Tris pH 7.0, 150 mM NaCl was recorded first. The final concentration of the cofactors, ArMs and Gst were 70 µM. The blank spectrum was subtracted from all sample spectra.

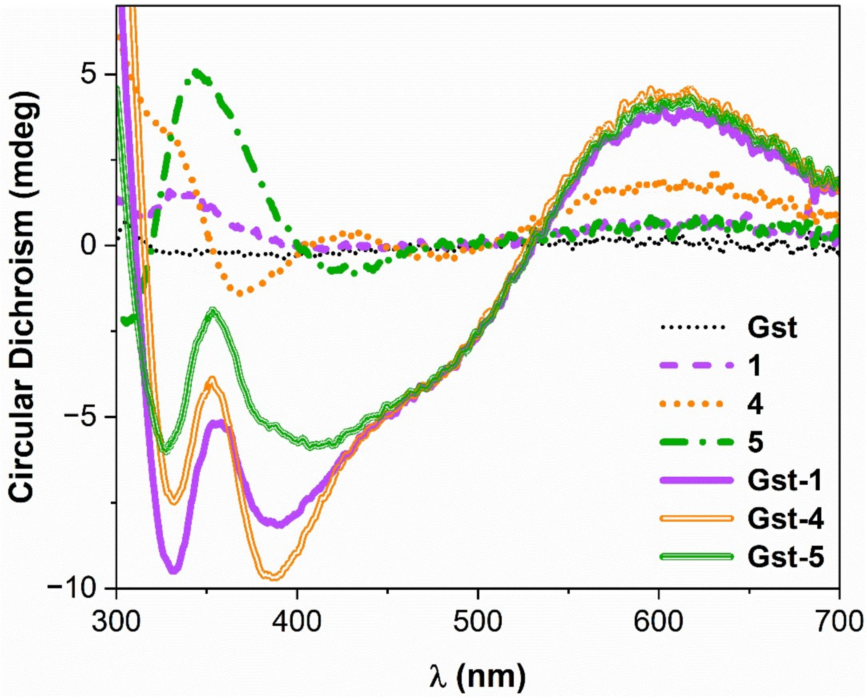

Fig. S4: Circular dichroism spectra of **1**, **4** and **5** in the presence or absence of Gst, with a control spectrum for Gst alone.

In the absence of Gst, the CD spectrum of cofactor **1** displays an azotochelin-based positive band between 330 nm and 335 nm and a very weak positive LMCT band at around 600 nm, consistent with a weak asymmetric induction effect exerted by the L-lysine backbone in the anchor unit of **1**. In contrast, the CD spectrum of Gst-**1**-ArM shows more intense features that resemble those previously reported for *Cj*CeuE-**1**-ArM, the crystal structure of which contained a Λ-configured Fe-centre and a partially-occupied Ir-centre with (*R*)-configuration (0.6 occupancy). [2]

Between ~450 nm and 700 nm, the CD spectra of Gst-**4**-ArM and Gst-**5**-ArM closely resemble that of Gst-1-ArM, indicating that all three ArMs contain a Λ-configured Fe-centre in the azotochelin-based anchor unit. Below 450 nm, however, where the spectral features of the azotochelin chromophore overlap with those of the respective Ir-based chromophores, differences are apparent. If compared with the Gst-**1**-ArM, the CD spectrum of Gst-**4**-ArM exhibits a more negative signal at around 380 nm and a more positive bands below 360 nm, whilst the Gst-**5**-ArM-based bands are less pronounced and reminiscent of those seen with cofactor **5**.

Taken together, the CD spectroscopic and catalytic results (Table S4) suggest that Gst-**1**-ArM, **4** and Gst-**4**-ArM contain the same enantiomer at Ir, which mainly produces (*R*)-**3**, with 28%, 77% and 95% e.e., respectively. In both Gst-**1**-ArM and Gst-**4**-ArM, productive interactions with the protein pocket contribute to the enantioselectivity. The chirality at the Ir-centre in Gst-**5**-ArM, on the other hand, is inverted and mainly determined by the chiral (*S*)-pyridinyl)propyl)benzenesulfonamide ligand in cofactor **5**, yielding (*S*)-**3** with 87% and 90% e.e., respectively.

2.17. Reductive release of the catalyst and reassembly of the CLArMAs

The reduction-triggered release of the siderophore-catalyst conjugate was conducted following a previously reported protocol [3] with specific modifications. Centrifuge tubes containing the recovered Gst-**1**-CLArMAs in storage buffer after the 8th cycle in the recycling studies were transferred to a nitrogen-filled bag containing an Eppendorf centrifuge and a shaker. Buffer (0.1 M MES / 0.5 M NaCl / pH 7.5), ddH_2O, weighed amounts of $Na_2S_2O_4$ and ferrozine, disposal and storage vials, as well as pipettes and tips, were also placed inside the bag, which was then sealed, and left overnight under N_2 flow. The next day, aqueous stock solutions of $Na_2S_2O_4$ (200 mg/mL) and ferrozine (3.6 mg/mL) were prepared in ddH_2O. The vials containing CLArMAs were centrifuged for 5 min at 4000 rpm, 4 °C, and the supernatant was carefully collected with a pipette and stored in a vial for analysis. Subsequently, a three-step process was executed: 1) 1920 µL buffer (0.1 M MES / 0.5 M NaCl / pH 7.5), and 60 µL ferrozine stock and 20 µL $Na_2S_2O_4$ stock were sequentially added to each vial containing CLArMAs, and

shaken for 5 minutes at 800 rpm. 2) The vials were centrifuged for 5 min at 4000 rpm, 4 °C. 3) The supernatants were carefully collected with a pipette and transferred to storage vials for analysis. The three-step process was repeated before the bag was unsealed. The recovered particles, denoted "Gst-**apo**-CLArMAs" were resuspended in 0.05 M Tris-HCl buffer, vigorously shaken, and centrifuged for 5 min at 4000 rpm, 4 °C. The supernatant was collected, and washing was repeated for another 2 times. The final recovered particles were subjected to a catalytic run to assess the effectiveness of catalyst release. Then, the particles were recharged with an alternative catalyst, **4**, as follows. Firstly, the recovered Gst-**apo**-CLArMAs were washed twice with buffer (0.05 M Tris-HCl / 0.15 M NaCl / pH 7.5). Then, 60 nmol (28 µL) of 2.174 mM **4**, and 472 µL of buffer were added to the reactor containing the recovered particles. The mixture was shaken for 1 h at 400 rpm, 20 °C, and then left under static conditions overnight at 4 °C. On the following day, the system was centrifuged for 5 min, 4000 rpm, and the recharged system, Gst-4-CLArMAs, rinsed with storage buffer before a reaction cycle was started. Following the exact same procedure, a triplicate of 200 µL of Gst-**1**-CLArMAs (not previously used in any reaction) went through the reduction steps and recharging with a third catalyst, **5**, to assembly Gst-**5**-CLArMAs, before testing for harmaline reduction.

Table S4. Catalytic activity towards the reduction of **2**: CLArMAs and control experiments with the respective free cofactor or ArM

Entry	Catalyst	e.e. %
1	**1**	racemic
2	Gst-**1**	28 (*R*)
3	Gst-**1**-CLArMAs	27.0 ± 0.7 (*R*)
4	**4**	77.0 ± 0.6 (*R*)
5	Gst-**4**	95.0 ± 0.1 (*R*)
6	Gst-**4**-CLArMAs	87.5 ± 0.5 (*R*)
7	**5**	87 ± 2 (*S*)
8	Gst-**5**	90 ± 2 (*S*)
9	Gst-**5**-CLArMAs	91.6 ± 0.5 (*S*)

3. AUTHOR CONTRIBUTIONS

A. H. M. was involved in conceptualisation, investigation, data curation, formal analysis, methodology, and writing – original draft. E. V. B. and S. A. T. were involved in methodology and investigation. K. S. W. was involved in conceptualisation, supervision and funding acquisition. G. G. was involved in funding acquisition and supervision. A.-K. D.-K. was involved in conceptualisation, supervision, project administration, funding acquisition and writing – editing and review. We thank the UKRI Engineering and Physical Sciences Research Council (EPSRC grant reference EP/T007338/1 and S. A. T. studentship EP/W524657/1) and the UKRI Biotechnology and Biological Sciences Research Council (BBSRC grant reference BB/W011131/1), for financial support.

4. REFERENCES

1. E. V. Blagova, A. H. Miller, M. Bennett, R. L. Booth, E. J. Dodson, A.-K. Duhme-Klair and K. S. Wilson, *Acta Crystallogr. D*, 2023, **79**, 694-705.
2. D. J. Raines, J. E. Clarke, E. V. Blagova, E. J. Dodson, K. S. Wilson and A.-K. Duhme-Klair, *Nat. Catal.*, 2018, **1**, 680-688.
3. A. H. Miller, E. V. Blagova, B. Large, R. L. Booth, K. S. Wilson and A.-K. Duhme-Klair, *ACS Catal.*, 2024, **14**, 3218-3227.
4. C. White, A. Yates, P. Maitlis and D. Heinekey, *Inorg. Synth.*, 1992, 228-234.
5. R. A. Sheldon, *App. Microbiol. Biotechnol.*, 2011, **92**, 467-477.
6. M. M. Bradford, *Anal. Biochem.*, 1976, **72**, 248-254.
7. A. Chimiak, R. Hider, A. Liu, J. Neilands, K. Nomoto, Y. Sugiura, A. Chimiak and J. Neilands, *Siderophores from microorganisms and plants*, 1984, 89-96.
